# Immediate Genetic Augmentation and Enhanced Habitat Connectivity Are Required to Secure the Future of an Iconic Endangered Freshwater Fish Population

**DOI:** 10.1111/eva.70019

**Published:** 2024-10-12

**Authors:** Alexandra Pavlova, Luke Pearce, Felicity Sturgiss, Erin Lake, Paul Sunnucks, Mark Lintermans

**Affiliations:** ^1^ Wildlife Genetic Management Group, School of Biological Sciences Monash University Melbourne Victoria Australia; ^2^ School of Biological Sciences Monash University Melbourne Victoria Australia; ^3^ NSW Department of Primary Industries Albury New South Wales Australia; ^4^ NSW Local Land Services, South East Local Land Services Braidwood New South Wales Australia; ^5^ NSW Department of Primary Industries, Department of Regional NSW Nowra New South Wales Australia; ^6^ Fish Fondler Pty Ltd Bungendore New South Wales Australia; ^7^ Centre for Applied Water Science, Institute for Applied Ecology University of Canberra Canberra Australian Capital Territory Australia

**Keywords:** effective number of breeders *N*_b_, effective population size *N*_e_, genetic diversity, genetic management, genetic monitoring, inbreeding, Macquarie perch *Macquaria australasica*, translocations

## Abstract

Genetic diversity is rapidly lost from small, isolated populations by genetic drift. Measuring the level of genetic drift using effective population size (*N*_e_) is highly useful for management. Single‐cohort genetic *N*_e_ estimators approximate the number of breeders in one season (*N*_b_): a value < 100 signals likely inbreeding depression. Per‐generation *N*_e_ < 1000 estimated from multiple cohort signals reduced adaptive potential. Natural populations rarely meet assumptions of *N*_e_‐estimation, so interpreting estimates is challenging. Macquarie perch is an endangered Australian freshwater fish threatened by severely reduced range, habitat loss, and fragmentation. To counteract low *N*_e_, augmented gene flow is being implemented in several populations. In the Murrumbidgee River, unknown effects of water management on among‐site connectivity impede the design of effective interventions. Using DArT SNPs for 328 Murrumbidgee individuals sampled across several sites and years with different flow conditions, we assessed population structure, site isolation, heterozygosity, inbreeding, and *N*_e_. We tested for inbreeding depression, assessed genetic diversity and dispersal, and evaluated whether individuals translocated from Cataract Reservoir to the Murrumbidgee River bred, and interbred with local fish. We found strong genetic structure, indicating complete or partial isolation of river fragments. This structure violates assumptions of *N*_e_ estimation, resulting in strongly downwardly biased *N*_b_ estimates unless assessed per‐site, highlighting the necessity to account for population structure while estimating *N*_e_. Inbreeding depression was not detected, but with low *N*_b_ at each site, inbreeding and inbreeding depression are likely. These results flagged the necessity to address within‐river population connectivity through flow management and genetic mixing through translocations among sites and from other populations. Three detected genetically diverse offspring of a translocated Cataract fish and a local parent indicated that genetic mixing is in progress. Including admixed individuals in estimates yielded lower *N*_e_ but higher heterozygosity, suggesting heterozygosity is a preferable indicator of genetic augmentation.

## 1 Introduction

Genetic diversity underpins species adaptation, survival, and long‐term persistence. However, this diversity is rapidly lost from small and isolated populations by genetic drift. This can lead to population extinction through reduced adaptive potential, chance loss of local adaptation, and reduced fitness of inbred individuals (inbreeding depression) (Harrisson et al. 2014; Frankham et al. 2019). The strength of genetic drift can be estimated by effective population size, *N*_e_—a metric that reflects the size of an ideal population with the same effects of genetic drift as in the target population. It also predicts effectiveness of selection relative to genetic drift (Charlesworth 2009). *N*_e_ can be estimated from genetic or demographic data, or approximated from census population size (Hoban et al. 2020; Hoban, Paz‐Vinas, et al. 2021). Global initiatives call for protecting and monitoring genetic diversity and effective population sizes (Hoban, Bruford, et al. 2021; Mastretta‐Yanes et al. 2024).

Recent guidelines provide useful *N*_e_ thresholds for predicting future population trajectories and triggering conservation genetic management: estimates of contemporary *N*_e_ (e.g., effective number of breeders in one season or year, *N*_b_) < 100 signal that inbreeding depression is likely, whereas per‐generation *N*_e_ < 1000 suggests that a population will lose genetic diversity by drift faster than it accumulates by mutation, indicating low capacity to adapt evolutionarily to environmental changes (Frankham, Bradshaw, and Brook 2014; Waples 2024a). *N*_e_ relevant to conservation thresholds for managing inbreeding depression and adaptive potential can be estimated from a single sample using linkage disequilibrium (LD) between pairs of selectively neutral unlinked loci. When estimated from a sample from a single cohort, an LD‐based estimate provides the effective number of breeders in one reproductive cycle (*N*_b_) relevant to inbreeding. When estimated from a mixed‐age sample, it provides average *N*_e_ per generation (i.e., per‐generation estimate) relevant for adaptive potential (Waples et al. 2013; Waples, Antao, and Luikart 2014). Many factors can affect *N*_e_, including number of breeding individuals, unequal sex ratio, variation in reproductive success, migration, genetic structure, and changes in population size (Charlesworth 2009; Waples 2024b). Thus, in populations where connectivity, breeding success, and population sizes are likely to depend on environmental conditions, genetic estimates of *N*_e_ could be challenging to interpret.

Traditional approaches to improve persistence of small threatened populations often aim at increasing population size via release of captive‐bred individuals, and/or enhancing physical connectivity to encourage dispersal and gene flow (Fraser 2008; Lowe and Allendorf 2010; Cramer et al. 2023). While both could successfully address genetic issues if performed adequately, the former could result in overall decrease of effective population size and population fitness if the number of breeders in the captive breeding program is limited. The latter could fail to provide adequate levels of gene flow to increase *N*_e_ and reduce genetic drift (Tringali and Bert 1998; Roques et al. 2018). Landscape genetic analysis can greatly facilitate management by estimating population structure, *N*_e_, *N*_b_, genetic diversity, and inbreeding and identifying barriers to dispersal and gene flow. Genetic augmentation—for example, through translocation of individuals or their gametes from one or multiple genetically more diverse and/or differentiated populations—presents an underused solution to rapidly enhance genetic diversity and population fitness (i.e., genetic rescue) (Ralls et al. 2018; Fitzpatrick et al. 2023). Importantly, genetic monitoring before and after augmentation enables tracking trajectories of wild populations, levels of recruitment, and success of management interventions. Methods for adjusting genetic augmentation through adaptive management are still in development, because monitored test cases are rare (Weeks et al. 2017; Hoffmann, Miller, and Weeks 2021; Lutz et al. 2021; Pavlova et al. 2023). This is partially due to slow acceptance by, or low capacity of, management agencies, and perceived high expense and under‐appreciation of the vital importance of managing genetic diversity for persistence of biodiversity (Pierson et al. 2016). Globally, genetic biodiversity has been in the Convention for Biological Diversity (CBD) since 1992, but only in 2022 CBD committed to setting targets, monitoring, and reporting (Hoban et al. 2022; Mastretta‐Yanes et al. 2024). Documenting examples of the benefits and cost‐effectiveness of genetic monitoring could accelerate acceptance and uptake of genetic management by wildlife managers with limited exposure to genetic approaches, and encourage collaborations with conservation geneticists, with beneficial outcomes for conservation of biodiversity.

An additional challenge to genetic management arises if a small population is also fragmented, because reduced gene flow among fragments can further accelerate genetic drift and disrupt other population processes such as mating systems, threatening population persistence (Amos et al. 2014; Radford et al. 2021). Understanding the degree of genetic drift and inbreeding within habitat fragments, and connectivity among them (including levels and directions of gene flow), is essential for designing appropriate genetic management solutions. This will help avoid harmful inbreeding, retain useful genetic diversity, and maintain adaptive potential, thus reducing extinction risk.

Macquarie perch (*Macquaria australasica*) is an at‐risk species for which genetic management is regarded as essential to prevent collapse of previously large, connected populations that are now small and isolated (Pavlova et al. 2017; Commonwealth of Australia 2018). It is an iconic freshwater fish species endemic to southeastern Australia. It is historically important for recreational fishing, and of significant cultural value for First Nations communities (Trueman 2011; Lintermans 2023a). Populations of Macquarie perch have declined significantly in recent decades due to habitat loss, degradation and fragmentation, overfishing, and the introduction of non‐native species (Commonwealth of Australia 2018; Lintermans 2023b). As a result, Macquarie perch is listed as a threatened species in all state jurisdictions within its historic range, and as endangered under the *Environment Protection and Biodiversity Conservation Act 1999* and the IUCN Red List (Lintermans et al. 2019).

Many remnant Macquarie perch populations were previously found to have low genetic diversity with an *N*_e_ below the threshold levels required to evolve adaptation and avoid harmful inbreeding (Pavlova et al. 2017). Accordingly, genetic augmentation has been recommended to increase genetic diversity, restore population‐level genetic health, and strengthen the potential of populations to adapt to changing environments (Pavlova et al. 2017). The national recovery plan for Macquarie perch adopted these recommendations (Commonwealth of Australia 2018), and implementation of genetic augmentation began in several populations. First reports on genetic monitoring of two Macquarie perch populations under genetic augmentation yielded two important lessons. First, fish that spent several generations in a lake environments might be less suitable for translocation to rivers compared to fish from riverine populations (Lutz et al. 2021). Second, physical connectivity moderated by river flow can promote recruitment success, whereas low flow and instream barriers can prevent upstream movement to spawning sites, leading to fewer breeders, and increased inbreeding and inbreeding depression (Pavlova et al. 2024).

A key Macquarie perch population requiring genetic augmentation is in the upper Murrumbidgee River. It is spatially the largest naturally occurring population in New South Wales (NSW) (Lintermans 2023c) and one of the largest nationally (occupying ~160 km of river), thus its conservation is of critical state and national importance. For over 60 years, > 90% of its natural flow has been regulated by damming and diversion to a hydroelectric scheme in an adjacent river system (Pendlebury et al. 1997; Snowy Scientific Committee 2010). The remaining flows in the upper Murrumbidgee are considered insufficient to submerge natural instream barriers to allow upstream movement of Macquarie perch, except in years of exceptionally high flow. Previous genetic studies using microsatellites found very low genetic diversity and *N*_e_ for this population, albeit these *N*_e_ estimates were based on geographically localized samples or very low sample sizes from three sites separated by potential low‐flow barriers such as cascades and extended steep gradient sections (Farrington, Lintermans, and Ebner 2014; Pavlova et al. 2017). Accordingly, genetic augmentation commenced in 2020 and 2022 through translocation to the upper Murrumbidgee of 139 wild individuals sourced from Cataract Reservoir (called Cataract Dam in earlier studies), a relatively genetically diverse population initiated ~100 years ago by translocation of mid/lower Murrumbidgee founders (Pavlova et al. 2017; Lutz et al. 2022). However, it is not clear how the presence of post‐damming instream low‐flow barriers in the upper Murrumbidgee may have influenced population genetic diversity and *N*_e_, how genetic estimates of *N*_e_ might be influenced if such barriers are ignored, and how management should account for these barriers during genetic augmentation. More comprehensive sampling of the Murrumbidgee Macquarie perch population across sites and years with different flow conditions was required to investigate population genetic structure, distribution of genetic diversity, and *N*_e_ across the river, where several instream barriers likely prevent movement and gene flow between breeding sites in some or all post‐impoundment river flow conditions.

Here we used a reduced‐representation (Diversity Arrays Technology [DArT]) genomic single‐nucleotide polymorphism (SNP) dataset for individuals sampled across several upper Murrumbidgee sites and years with different flow conditions to assess population structure and the degree of isolation between river fragments, genetic diversity, the level of inbreeding, and the number of breeding Macquarie perch individuals (*N*_b_) within each site. We also test whether habitat (approximated by site), environmental variation (approximated by birth year), and genetic diversity influence juvenile individual growth: significant positive heterozygosity–growth correlations might be expected under inbreeding depression, as suggested for another riverine Macquarie perch population (Pavlova et al. 2024). We also tested whether genetic diversity in the Murrumbidgee River increases from upstream to downstream, as would be expected under downstream dispersal and impeded upstream dispersal resulting from natural instream barriers and low environmental flows (Lintermans 2023c). Finally, we evaluated whether the individuals translocated from Cataract Reservoir to the Murrumbidgee River bred, and interbred with local fish. Providing these estimates for a riverine Macquarie perch population will serve as an example of how to build an understanding of management requirements and inform genetic management including to what extent a given population can be an appropriate source of migrants for genetic rescue of other populations (Pavlova et al. 2017, 2024). Understanding how population structure and genetic augmentation may influence genetic estimates of effective population size can provide insights on how to improve genetic management and monitoring of other species.

## 2 Methods

### 2.1 Study System

**FIGURE 1:**
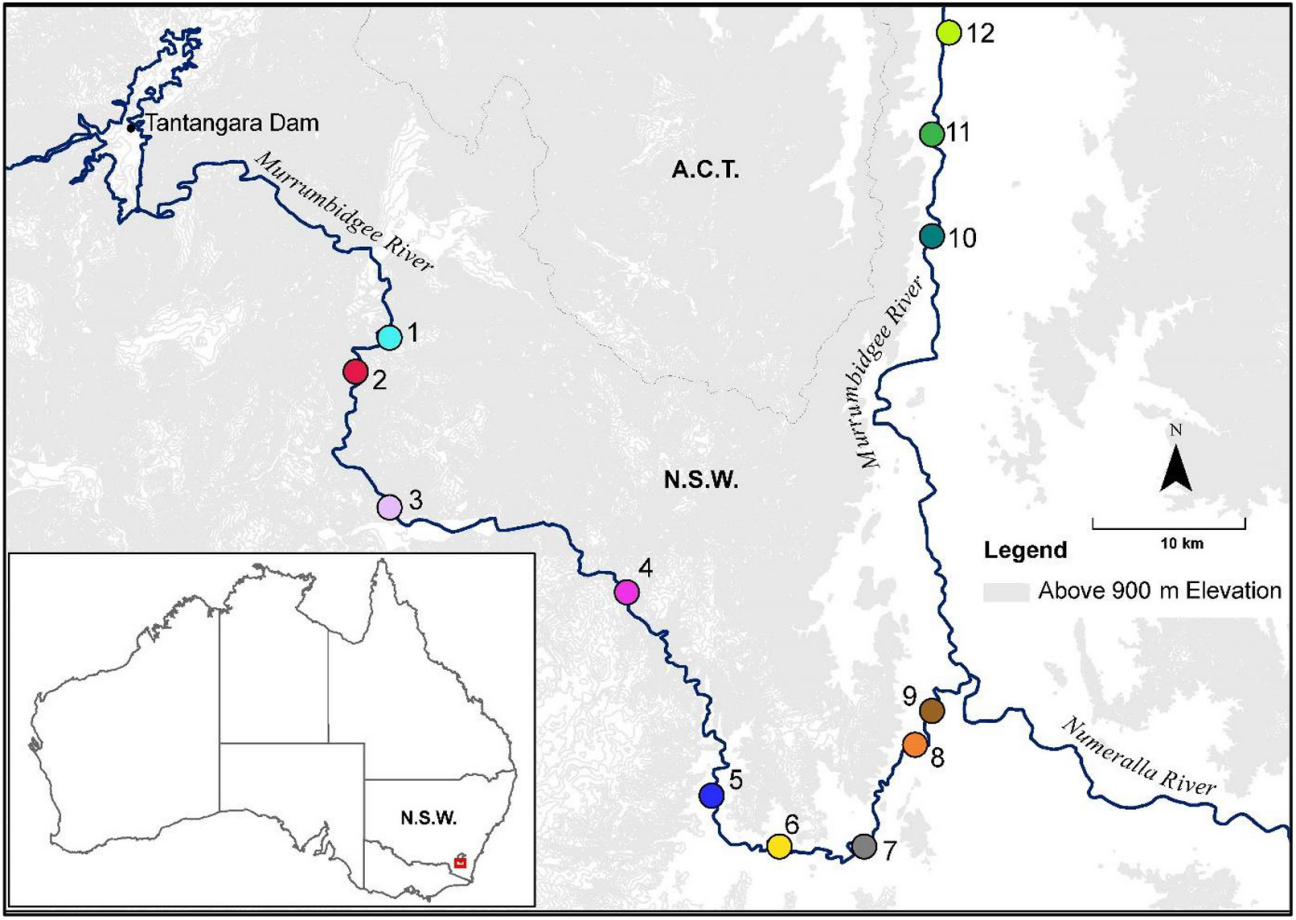
Map of sampling sites (colored circles) for the upper Murrumbidgee River Macquarie perch population. Sites are numbered from upstream to downstream (sample sizes in Table 1). Colors of the circles correspond to colors on Figures 2, 3, 4, and Appendix C: Figures C1–C4 in Supporting Information. On the inset, the red rectangle shows the location of the study area within Australia. The hypothesized gene flow between sites in the Upper Murrumbidgee could be represented as 1|→2?→3|→4?→5←→6←→7?→8←→9|→10?→11?→12, where a putative barrier to gene flow (cascade/waterfall/gorge) is denoted as “|”, downstream, but not upstream movement as “→,” bidirectional connectivity as “←→,” and suspected impeded upstream gene flow as “?.” These barriers to upstream gene flow (potentially partial and/or varying by year/flow) were hypothesized based on visual assessment or remote sensing of flow velocity and gradient, and have not been tested by published fish tagging or movement studies, although some fish movement in the lower recruitment reaches was observed (P. Haantjens, unpublished data).

The Murrumbidgee River is the second longest Australian river. Approximately 65 km downstream of its source it is impounded by Tantangara Dam (Figure 1). This dam, constructed in 1960, is operated to divert 90%–99% of Murrumbidgee flows to the adjacent coastal Snowy River catchment, and has spilled into the Murrumbidgee River (emulating natural high flows) only three times since its construction (in 1962, 1975, and 1992; Pendlebury et al. 1997). Only small volumes of water were released from the dam to the Murrumbidgee until 2011, from when larger environmental flows have been released, with maximum daily release volumes of 1500 ML (Snowy Scientific Committee 2010; Lintermans 2023d). The present study was conducted in the ~160‐km stretch of the upper Murrumbidgee River starting ~30 km downstream of Tantangara dam (Figure 1). This river segment has a channel consisting of rocky bedrock and boulder substrates in a deeply incised valley with numerous cascades and gorges. Inflow from tributaries restores the stream to ~27% of average natural flow at Site 7 and ~54% downstream of Site 9 (Figure 1) (Pendlebury et al. 1997).

### 2.2 Species Biology and Barriers to Movement

Macquarie perch *Macquaria australasica* is a moderate‐sized, iteroparous long‐lived southeastern Australian freshwater percichthyid fish (Lintermans 2023b). Males reach sexual maturity at ~150–210 mm length and females at ~300 mm (Lintermans 2023b). Upstream spawning migrations are well described for impoundment populations (Tonkin et al. 2018) but are less understood in rivers (Koster et al. 2013). In the upper Murrumbidgee River, spawning migrations of tens of kilometres downstream and upstream have been recorded in river reaches with no impassable barriers (P. Haantjens, unpublished data). Spawning aggregations in key spawning areas have been reported in several river populations (Kearns et al. 2012; Tonkin et al. 2016). Females lay adhesive, demersal eggs at the foot of pools subsequently to lodge in downstream riffles, or directly in riffles, where males externally fertilize them (Cadwallader and Rogan 1977; Tonkin, Lyon, and Pickworth 2010; Tonkin et al. 2016; Broadhurst et al. 2019). No parental care is provided (Humphries, King, and Koehn 1999; Growns 2004).

The upper Murrumbidgee River currently holds one of the largest self‐sustaining natural Macquarie perch populations (Lintermans 2023c), with mid/lower Murrumbidgee populations declining significantly or locally extinct since the mid‐1960s as a result of sedimentation, land clearing, barriers to fish passage, flow regulation including cold‐water discharge, overfishing, and interaction with alien species (Lintermans 2002; Lintermans et al. 2019). The upper Murrumbidgee River population was surveyed at 12 sites spanning 157 km, focusing on 90 km of recruitment reach between Sites 1 and 9 (Figure 1, sites are numbered from upstream to downstream). Macquarie perch is absent upstream of Site 1. There are no self‐sustaining populations known below Site 9. A number of natural and anthropogenic barriers are thought to prevent or restrict upstream movement of Macquarie perch within the recruitment reach (Sites 1–9), including cascades upstream of Site 1, between Sites 1–2, 3–4, and 4–5, and a weir between Sites 7–8 with a partially effective fishway providing some upstream movement (ML, unpublished data). Gorges and waterfalls downstream of the recruitment reach may also restrict fish movement between Sites 9 and 12 (Figure 1). By exposing instream barriers, low flow is known to restrict fish passage to breeding grounds and impede breeding in the Murrumbidgee River tributary, the Cotter River (Broadhurst, Clear, and Lintermans 2016; Pavlova et al. 2024). The period of intensive sampling for this study (2020–2023) occurred during a prolonged La Niña event with high rainfall and above‐average flows, which followed a major drought in NSW in 2017–2019. The mean daily flow was lowest in 2018 and 2019 (92 and 74 ML, respectively at Site 2) and highest in 2022 (678 ML at Site 2; Appendix A: Table A1 in Supporting Information). Thus, we expected the lowest number of breeders in the upper Murrumbidgee in 2019—the year of lowest flow—followed by 2018. While high flows should facilitate movement of adult fish past instream barriers to their breeding grounds, very high flows immediately following the Macquarie perch spawning season in November to mid‐December can wash out eggs and damage developing larvae (Tonkin et al. 2017) and high flows in 2022 are thought to have resulted in recruitment failures of the 2022 birth‐year cohort in the upstream sites (Table A1 in Supporting Information) (Lintermans 2022).

### 2.3 Genetic Management

To rectify low genetic diversity and strong inbreeding (*F* > 0.45; Pavlova et al. 2017) of the upper Murrumbidgee population, genetic augmentation was implemented, through translocation from the Cataract Reservoir population translocated ~100 years ago from the mid/lower Murrumbidgee. A total of 139 Cataract Reservoir Macquarie perch were translocated in November 2020 and May 2022 to Sites 5, 7, and 8 (details in Table A1 in Supporting Information). Here we assess reproductive success of the potential Cataract breeders.

### 2.4 Sampling

Intensive sampling in the upper Murrumbidgee River occurred in 2020–2023 and focused on the recruitment reach, Sites 1–9 (details in Lintermans 2023c); fish sampled from 2002 to 2019 and in downstream Sites 10–12 were also included (Table 1, Table A2 in Supporting Information). Two sentinel sites—2 and 8—were sampled each year in 2020–2023 (Lintermans 2023b). Given recorded mobility of Macquarie perch of many kilometers per day, including upstream (Broadhurst et al. 2013; Thiem et al. 2013; Tonkin et al. 2018), groups of individuals collected from each of Sites 1–9 should be representative of larger habitat units, potentially constrained by upstream barriers at some sites. Individuals collected from Sites 10 to 12 likely represent dispersers from upstream breeding sites, or offspring from rare, sporadic, local breeding events.

**TABLE 1:** Summary of Macquarie perch samples, captured in each *sampling year* in each sampling site (except sampling years 2002–2019 are combined here, but see Table A2 in Supporting Information).

Sampling sites	Stream distance from Site 1	Lat	Long	Sampling year categories					Sample size
				2002–2019	2020	2021	2022	2023	
Site 1	0.0	−35.87	148.81					9	9
Site 2*	5.0	−35.89	148.79		29	48	9	7	93
Site 3	18.3	−35.97	148.81			31	7		38
Site 4	37.0	−36.02	148.95					8	8
Site 5	60.5	−36.14	149.00	18		19			37
Site 6	69.3	−36.17	149.04					12	12
Site 7	77.5	−36.17	149.09					3	3
Site 8*	88.3	−36.11	149.12	2	14	37	18	30	101
Site 9	90.3	−36.09	149.13	7		9	2	7	25
Site 10	133.8	−35.81	149.13	1					1
Site 11	141.3	−35.75	149.13	1					1
Site 12	156.8	−35.69	149.14	2					2
Total				31	43	144	36	76	330

*Note:* Sites are numbered from the most upstream (Site 1) to the most downstream (Site 12, Figure 1). Two sentinel sites are marked with asterisks.

Each sampling site spanned 200–500 m of boat‐accessible river length. Within each site, sampling occurred in pool habitats > 0.5 m depth where nets could be deployed effectively from a boat. Fish caught are considered a random representation of individuals at a site, because the nets were widely distributed across the entire pool length at each site and the net types used catch a broad range of size classes (Lintermans 2016, 2023a). After total length was measured and a genetic sample (fin clip) taken, individuals were released at the site of capture. Genetic samples were preserved in 100% ethanol and stored −20°C at the conclusion of each field trip. To estimate the ancestry of Murrumbidgee individuals captured after translocation from Cataract Reservoir, we included samples of Cataract Reservoir individuals as a reference (Pavlova et al. 2024). A total of 330 samples from Murrumbidgee and 67 from Cataract Reservoir were used in this study (Table A2 in Supporting Information).

### 2.5 Identifying Birth‐Year Cohorts

For individuals collected from 2020 onward, total length was used to estimate age and birth‐year. Individuals < 90 mm formed a distinct peak on the length distribution of all fish, and were assigned to young‐of‐year (0–1YO), based on an observed breakpoint in empirical distribution of lengths (Appendix B: Figure B1 in Supporting Information). There were no clear breaks between remaining peaks of the combined length distribution on the plot of all fish, indicating year‐to‐year variation in growth rates. When length distributions were examined for each sampling year, consistent breaks were observed between 1 and 2YO and older fish (Figure B2 in Supporting Information), but the length range was larger in the 2020 sample compared to the 2021–2023 samples. Accordingly, we assigned fish of 100–210 mm sampled in 2020 and fish of 100–180 mm sampled in 2021–2023 to 1–2YO. Accurate identification of age 0–1YO and age 1–2YO individuals based on length is widely used and accepted for this species (Broadhurst et al. 2013; Lintermans 2013, 2016; Tonkin et al. 2017). For larger/older individuals, we assigned fish of 211–270 mm sampled in 2020 and fish of 181–270 mm sampled in 2021–2023 to 2–3YO. All individuals > 271 mm were assigned to > 3YO (i.e., “adults”). Given the threatened status of Macquarie perch, lethal sampling for otolith analysis for confident aging was not desirable. The age categories applied here are supported by previous studies that have investigated otolith‐based age–length relationships (Tonkin et al. 2014, 2018; Todd and Lintermans 2015). While it is possible that some individuals aged as 2–3YO are in fact older, they comprise only 7.3% of all aged samples (most belonging to the 2020 cohort, where they represent only 12.4% of samples), thus potential errors should not strongly influence our conclusions.

Capture dates of individuals assigned to age groups 0–1YO, 1–2YO, and 2–3YO were used to assign individuals to six birth‐year cohorts, 2017–2022, with sample sizes ranging from 1 (2017) to 144 (2020) (Table 2). All but three of 25 measured fish sampled before 2020 were > 271 mm (> 3YO according to our criteria). We analyzed all individuals sampled before 2020 and more recently collected > 3YO fish together as a category “adults” (Table 2).

**TABLE 2:** Number of analyzed Murrumbidgee genotypes for adults (collected 2002–2023) and *each cohort* of juveniles across seven Murrumbidgee River sampling sites, numbered from the most upstream (Site 1) to most downstream (Site 12, Figure 1).

Sampling site	Number of adults	Cohorts of juveniles						Total
		2017	2018	2019	2020	2021	2022	
Site 1	6				3			9
Site 2	7		24	25	35	1		92
Site 3	1		1	2	33	1		38
Site 4	1				7			8
Site 5	18			7	12			37
Site 6	4				3		5	12
Site 7	2						1	3
Site 8	14^a^	1	6	6	47	3^b^	23^b^	100
Site 9	17				4	3^b^	1	25
Site 10	1							1
Site 11	1							1
Site 12	2							2
Total	74	1	31	40	144	8	30	328

*Note:* Only the 182 fish from cohorts 2020–2022 were born after Cataract translocations started (shaded columns).

^a^ Group including a recaptured fish of Cataract Reservoir origin.

^b^ Groups including admixed fish.

### 2.6 Size‐at‐Age Residuals

Documenting inbreeding depression in wild populations relies on the ability to collect fitness data (such as lifetime reproductive success), which is logistically challenging and takes many years or decades for long‐lived species (Harrisson et al. 2019; Zilko et al. 2020). In some cases, growth rate of individuals, which is relatively easy to estimate, can be an indicative fitness component (Lutz et al. 2021). For 257 Macquarie perch individuals assigned to cohorts 0–1YO, 1–2YO, and 2–3YO, size‐at‐age residuals from the Gompertz growth model were calculated, to be used as a response variable in inbreeding depression models. Initial parameters were *b*1 = 200, *b*2 = 0.5, and *b*3 = 0.3 (Appendix B in Supporting Information).

### 2.7 Sequencing, Genotyping, and Filtering

A commercial provider DArT generated reduced‐representation genomic sequencing datasets. SNP genotypes were obtained by co‐analyzing new Murrumbidgee samples and previously analyzed Cataract Reservoir samples (Lutz et al. 2022; Pavlova et al. 2024). The Cataract Reservoir genotypes were used to create a reference for detecting admixed offspring resulting from interbreeding of Murrumbidgee and translocated Cataract Reservoir individuals.

Genotypes were filtered to remove loci with mean read depth < 6 and > 50, loci with reproducibility < 95%, loci missing in > 10% of individuals, individuals with > 20% of missing data. We also removed loci that were likely to represent a technical artefact—with heterozygosity significantly higher than 0.5 in either Murrumbidgee (analyzed as a whole) or Cataract Reservoir—using function *filter.excess.het.R* (Robledo‐Ruiz et al. 2023). One random SNP per DArT‐tag was retained, to reduce loci that are highly nonindependent through very close physical linkage. Unless noted otherwise, genotypes were filtered using *dartR v2.0.4* (Mijangos et al. 2022) in R v4.3.0 (R Core Team 2021) with RStudio v2023.03.1+446 (RStudio Team 2020). The final complete dataset comprised 3447 biallelic SNPs scored for 375 individuals (328 Murrumbidgee and 47 Cataract Reservoir) with 0.99% missing data. This dataset (and its subsets) was used for assessment of genetic diversity.

Because including physically linked loci could bias results of analyses that assume independent inheritance of loci (e.g., analyses of sibship, admixture, *N*_e_, and recent migration rates), we also created datasets where SNP loci were thinned based on physical proximity. Applying LD‐based filters was not appropriate, because Murrumbidgee Macquarie perch previously showed signs of inbreeding, so important signal could be lost by such filtering. We thus conducted LD decay analysis to find the physical distance between loci beyond which LD (*r* ^2^) falls to background levels (50 Kb), using SNP‐containing DArT tags mapped to the draft Macquarie perch genome (Pavlova et al. 2022) for an adults‐only dataset, using PopLDdecay (Zhang et al. 2018). To reduce physical linkage, we then removed unmapped loci from the datasets of adults and all Murrumbidgee individuals and thinned loci within 50 Kb windows using *SambaR* (de Jong et al. 2021) and *dartR* R packages. The adult dataset (72 individuals scored for 1607 SNPs) was used to run PCoA and admixture analyses and calculate *F*_ST_‐values in *SambaR*. The dataset for all individuals of Murrumbidgee ancestry (325 individuals scored for 2626 SNPs, excluding the single recaptured Cataract Reservoir individual) was used for calculation of recent immigration rates and *N*_e_. For sibship analyses, loci with a minimum count of minor allele < 2 were also removed, and the data were further split into upstream (Sites 1–4; 145 individuals scored for 964 SNPs) and downstream (Sites 5–12; 180 individuals scored for 1242 SNPs) sub‐datasets (details in Appendix C in Supporting Information).

### 2.8 Genetic Structure

To assess whether any of the sampled Murrumbidgee individuals had Cataract Reservoir or admixed ancestry, principal coordinate analysis (PCA) was conducted on the complete dataset, implemented by *dartr* function *gl*.*pcoa*. Because Murrumbidgee and Cataract Reservoir fish are genetically distinct even on a less‐resolving microsatellite analyses (Pavlova et al. 2017), we expected that one of the first two PCA axes would differentiate these populations as distinct genetic clusters, and that individuals with intermediate values of the relevant PC axis would represent admixed Murrumbidgee x Cataract offspring. We also ran PCA on all samples of Murrumbidgee ancestry, to test for presence of within‐population structure.

To ensure that population structure detected from all individuals reflects the effects of barriers to dispersal/gene flow rather than temporary family structure due to clustering of pre‐dispersal juveniles, or simply isolation‐by‐distance, we analyzed genotypes of fish classified as “adults” using wrapper functions of *SambaR* to run PCA, admixture analysis in R package *LEA* (Frichot et al. 2014) and calculate among‐sites *F*_ST_‐values. We used *F*_ST_‐values and river‐distances to test for isolation‐by‐distance using Mantel test in *dartR*. We did not test for isolation‐by‐barriers, because data on all instream barriers to Macquarie perch movement were lacking, precluding modelling their effect. Finally, to test for asymmetric downstream dispersal, we estimated recent (1–2 generation) immigration rates among sampling sites, and among groups comprising likely genetic clusters, using BayesAss3 (Wilson and Rannala 2003) implemented in *BA3‐SNPs* (Mussmann et al. 2019); individuals of admixed ancestry were excluded (details in Appendix C in Supporting Information).

### 2.9 Genetic Diversity and Inbreeding

To assess individual heterozygosity, we calculated proportion of heterozygous sites (PHt), for the complete dataset of 3447 SNPs assessed for all 375 individuals (from the Murrumbidgee and Cataract Reservoir), and for the dataset of 3173 SNPs assessed for 328 individuals of all ancestries captured in the Murrumbidgee, using *dartR*. We tested whether heterozygosity increases linearly with distance from the most upstream Site 1, using a linear model built with the *lm* function of R package *stats*. We also tested whether heterozygosity differed significantly across ancestries (i.e., Murrumbidgee, Cataract, admixed; using values from the complete dataset), sites, cohorts/adults, and cohort‐per‐site groups for groups of > 10 individuals (using values from the Murrumbidgee dataset) using analysis of variance (ANOVA), fitted using the *aov* function of package *stats* in R. We tested for difference between groups using post hoc Tukey Honest Significant Differences (HSD) test implemented in *TukeyHSD* function.

For the 250 juveniles and subadults (e.g., fish < 3YO) of Murrumbidgee‐only ancestry born from 2018 to 2022, we assessed whether variance in heterozygosity is significantly explained by population structure, birth years, or both. To do that, we fitted linear models, with site, cohort, site and cohort, and site, cohort, and their interaction as predictors of individual PHt. ANOVA was used to compare models and determine whether adding another predictor significantly improved the model fit.

Inbreeding is a relative measure, indicating how inbred a particular population is relative to a related outbred population (likewise for individuals). If the Cataract Reservoir population is assumed to be outbred based on having a large estimated population size and accompanying high genetic diversity (Pavlova et al. 2017), then the effective inbreeding coefficients of the Murrumbidgee samples can be calculated from expected heterozygosity (*H*e) as *F*_e_ = 1—*H*e_inbred_/*H*e_outbred_ (Frankham 1998). We estimated population values of observed (*H*o) and expected heterozygosity for the pooled Murrumbidgee (*N* = 326) and pooled Cataract Reservoir (*N* = 45) samples, using function *gl.report.heterozygosity* method = “pop,” n.invariant = 0. We also estimated population heterozygosity for each cohort, each site, and each site‐per‐cohort with *N* > 12, and for four pooled groups of site‐per‐cohort samples (Sites 2 + 3 and Sites 7 + 8 + 9 were merged based on low differentiation/genetic clustering). To ensure comparability of values across groups, calculations of population heterozygosity used a dataset for 3068 SNPs variable in at least one individual captured in the Murrumbidgee River (regardless of origin).

### 2.10 Effective Population Size

We estimated *N*_e_ using a single‐sample LD‐based method implemented in *LDNe* (Waples and Do 2008; Do et al. 2014) using the *gl.LDNe* wrapper in *dartR* ignoring alleles with frequency < 0.01 (Pcrit = 0.01) and using confidence intervals calculated by method JackKnife on samples. For an iteroparous species such as Macquarie perch, LD‐based ${\hat{N}}_{e}$ estimated from a single cohort (${\hat{N}}_{b}$) represents the harmonic mean of the effective number of breeders in one reproductive cycle (short‐term *N*_b_) and the effective population size per generation (per‐generation *N*_e_), but this can be adjusted to represent true *N*_b_ using formula (8) of Waples, Antao, and Luikart (2014): adjusted *N*_b __adj_ = ${\hat{N}}_{b}$/(1.26–0.323 × *N*_b_/*N*_e_) when the ratio *N*_b_/*N*_e_ is known or can be predicted from two or three life history traits (Waples et al. 2013). For Macquarie perch this ratio was estimated earlier from two traits—adult life span (AL = 23 years) and age at maturity (*α* = 3 years)—as *N*_b_/*N*_e_ = 0.485 + 0.758 log × (AL/*α*) = 1.156 (Pavlova et al. 2017), thus according to the above formula, *N*_b__adj_ = ${\hat{N}}_{b}$/0.887. When per‐cohort short‐term *N*_b_ estimates are available, per‐generation *N*_e__adj_ can be estimated by dividing *N*_b __adj_ by *N*_b_/*N*_e_ (Waples, Antao, and Luikart 2014), so *N*_e __adj_ = *N*_b__adj_/1.156 for Macquarie perch. Alternatively, the LD‐based estimate ${\hat{N}}_{e}$ from a mixed‐age sample should approximate per‐generation *N*_e_; although downwardly biased, it is least biased when *N*_b_/*N*_e_ ~ 1 and when the number of cohorts in a sample is close to the length of the generation time (Waples, Antao, and Luikart 2014). Although for *N*_b_ < 50 biases can be large, their magnitude would not influence management decisions (Luikart et al. 2021). Because generation time for Macquarie perch is ~7 years and *N*_b_/*N*_e_ ~ 1 (Pavlova et al. 2017), ${\hat{N}}_{e}$ from a sample including five known cohorts and adults from additional cohorts could represent a reasonable proxy for per‐generation *N*_e_.

Genetic estimates of *N*_e_ can be biased by factors affecting LD in a population, such as the presence of population structure, population size dynamics over time, and inclusion of migrants from another population or first generation “hybrids” from different sources (Pavlova et al. 2024; Waples 2024b). To test how single‐year and per‐generation *N*_e_ estimates from real‐life monitoring data change depending on presence of population structure, pooling different cohorts, and including admixed and translocated individuals, we used *LDNe* to calculate short‐term ${\hat{N}}_{b}$ and per‐generation ${\hat{N}}_{e}$ for four categories of groups with > 10 samples per group: (1) *N*_b_ per cohort per site and *N*_e_ for adults (11 groups); (2) *N*_b_ per cohort, all sites pooled (5 groups); (3) *N*_e_ per site, cohorts and adults pooled (8 groups); and (4) per‐generation *N*_e_ for all data. Groups that included admixed individuals were analyzed with and without them. In addition, we estimated ${\hat{N}}_{b}$ per cohort for two groups of sites that belonged to the same genotype cluster, and thus are likely connected by gene flow (Sites 2–3 and 7–9). Due to strong population structure and low cohort‐per‐site replication, we did not test for the effect of river flow on heterozygosity or ${\hat{N}}_{b}$ estimates.

### 2.11 Identity and Sibship Analyses

Analyses of identity and sibship were run in *Colony2* v 2.0.7.0 (Jones and Wang 2010) assuming codominant markers, allelic dropout rate 0.005, other error rate 0.001, dioecious, diploid, polygamous individuals, and inbreeding. The analysis employed the most accurate full‐likelihood method using default high precision, medium run length, while updating allele frequencies. Full sibship‐scaling was implemented with no prior sibship assumed. Five replicate runs were performed for each analysis by setting different random seeds. Identical genotypes and full‐sib families were accepted if they were detected by at least three replicates. Cases of two or more identical genotypes (i.e., the same individual sampled twice, or matching siblings) and full‐sibship assignments were used to infer dispersal patterns. To explore temporal connectivity between some sites, we examined which cohorts were involved in cases of apparent dispersal among genetic groups (as defined by PCA).

### 2.12 Testing for the Effect of Site, Cohort, and Heterozygosity on Size‐at‐Age Residuals

For the 250 juveniles of Murrumbidgee‐only ancestry born from 2018 to 2022, we explored variance in size‐at‐age residuals in space and time. We built linear models to test whether site, cohort, site and cohort fitted as main effects, or site, cohort, and their interaction explained significant proportions of variance in size‐at‐age residuals. Significance of the pairwise differences between sites and cohorts was tested for by fitting ANOVA followed by post hoc Tukey testing (when ANOVA was significant at *p* < 0.05). As above, ANOVA was used to determine whether the model fit was significantly improved after adding a predictor and so to identify best‐fit models. To test whether individual heterozygosity explained additional variance in juvenile growth after all significant covariates were accounted for (i.e., whether there was evidence for inbreeding depression), we fitted PHt as an additional predictor to those used in the best‐fit model. Faster growth could be expected to represent stronger fitness in predator‐rich environments such as the upper Murrumbidgee catchment where alien salmonids are abundant and stocked regularly (NSW DPI 2024). Significant positive relationship between PHt and growth rate (i.e., expressions of inbreeding depression) might be limited to the stressful low‐flow conditions of years 2018–2019.

## 3 Results

### 3.1 Population Genetic Structure

**FIGURE 2:**
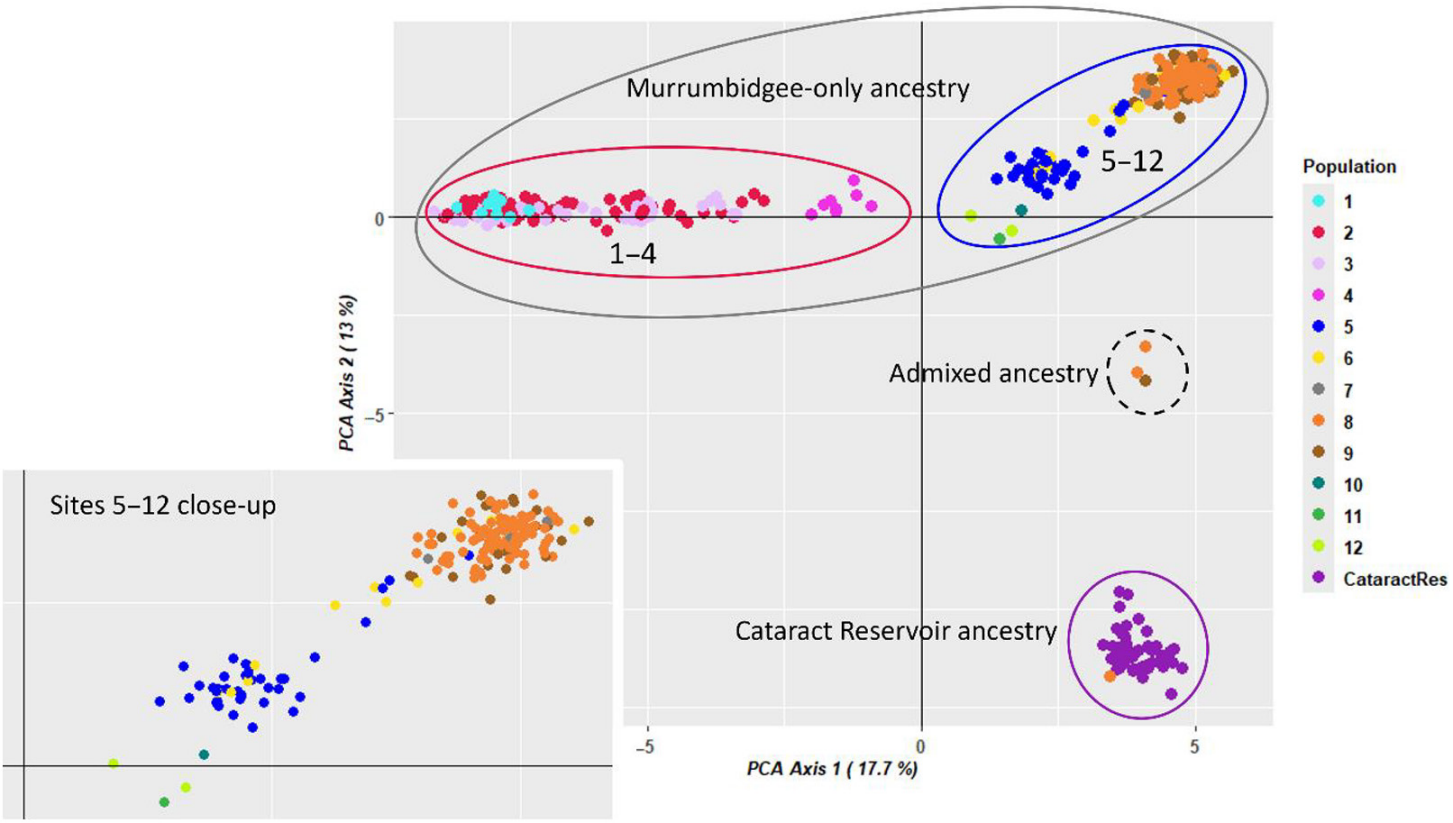
First two axes of the principal coordinate analysis (PCA) on Murrumbidgee and Cataract Reservoir samples. Ovals encompass relevant ancestries or sites. Sites are color‐coded as per legend and Figure 1.

The first principal component (PC1) of the PCA of Murrumbidgee and Cataract Reservoir genotypes explained mainly structure within the Murrumbidgee, where individuals from the upper reach Sites 1–4 (PC1 < −0.8; red oval on Figure 2) clustered separately from the lower reach Sites 5–12 (blue oval). Meanwhile, PC2 of the same PCA showed a clear separation between Murrumbidgee (PC2 > −2; grey oval) and Cataract Reservoir (PC2 < −8; purple oval) individuals, and enabled detection of one individual of Cataract Reservoir ancestry (orange dot within the purple oval) and three individuals of admixed ancestry (dashed oval) among Murrumbidgee individuals. The Cataract‐ancestry fish sampled in the Murrumbidgee must be a recaptured fish translocated in 2020, because it was too large to be an offspring of two Cataract translocatees. The three fish of admixed Murrumbidgee x Cataract Reservoir origin were captured at Sites 9 and 8, with their size suggesting that two were born in 2021 and one in 2022. All three were caught in 2023, 3 years after the initial translocation, and must be offspring of one translocated and one local parent. This indicates that Cataract fish bred at least twice in the Murrumbidgee, in 2021 and 2022.

**FIGURE 3:**
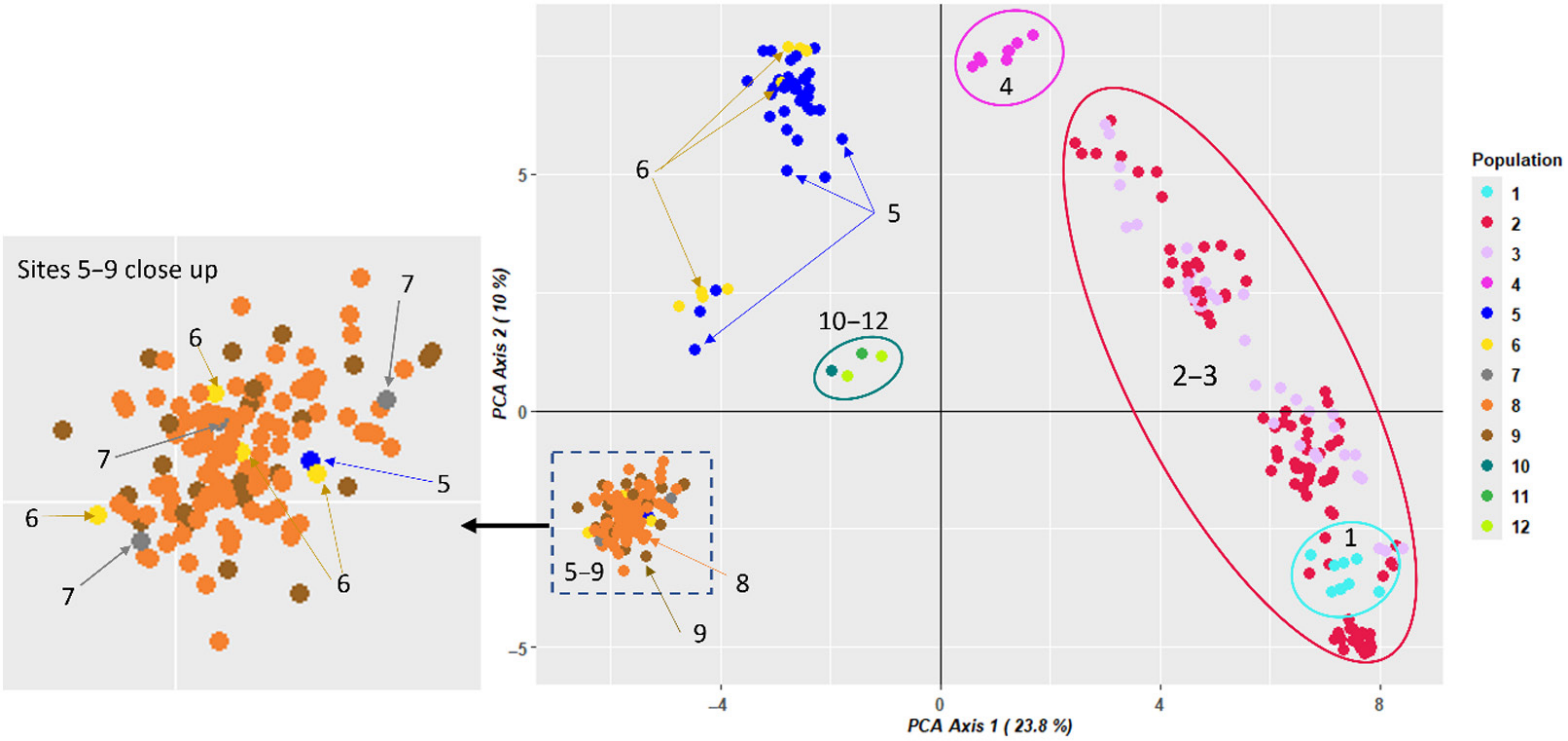
First two axes of the PCA on Murrumbidgee‐only samples (excluding individuals of Cataract Reservoir and admixed ancestry); ovals encompass sampling sites, inset shows a close‐up for a genetic group comprising Sites 5–9. Sites are color‐coded as per legend and Figure 1 (see Figure D1 in Supporting Information for Axes 1–2 plot with individuals colored by membership in 12 largest full‐sib families and Figure C2 in Supporting Information for the plot for adults).

PC1 from the PCA of the Murrumbidgee‐only samples (excluding the admixed individuals; Figure 3), further distinguished apparently connected upper reach Sites 2–3 (red oval, Figure 3) with at least occasional connectivity between Sites 1 (light blue oval) and 2, from apparently isolated site 4 (magenta oval) and all downstream Sites 5–12 (PC1 < 0). Both PC1 and PC2 further distinguish four clusters within the lower reach. The geographically intermediate site 5 (−5.3 < PC1 < −1.7; dark blue oval), while apparently isolated from the upper reach, appeared to be somewhat genetically connected to other sites of the lower reach. In particular, tight genetic clustering of all individuals from Sites 7–9 with four individuals from Site 6 and an individual from Site 5 (inset on Figure 3; PC1 < 0, PC2 < 0), suggests that occasional upstream dispersal from Sites 7 to 6 and from 6 to 5 is possible. Most individuals from Site 5 (*N* = 33; dark blue dots) and four from Site 6 (yellow dots) clustered separately (PC1 < 0, PC2 > 4.5), suggesting limited gene exchange between Sites 5 and 6 and more downstream sites. Meanwhile the remaining three individuals from Site 5 and four from Site 6 comprised a genetically intermediate cluster (PC1 < −3, 0 < PC2 < 3), likely representing offspring of parents from the former two clusters. This structure, and the presence of occasional gene exchange between Site 5 and downstream Sites 7–9 of the recruitment reach, were also detected by the PCA and admixture analyses of adults (Appendix C: Figures C2 and C4 in Supporting Information). The very small number of fish from Sites 10 to 12 (*N* = 4) precluded making inferences about the relationships of fish from these sites. Isolation‐by‐distance was significant, explaining ~39% of variance in *F*_ST_ values (Mantel test *r* = 0.62, *p* = 0.001); however, genetic distances at short inter‐site geographic distances were highly variable (Figure C7 in Supporting Information), and the cross‐entropy criterion calculated by *LEA* suggested that at least three distinct genetic clusters are present (Figure C3 in Supporting Information). Together, these results indicated that both isolation‐by‐distance and barriers to dispersal shaped the observed population structure.

### 3.2 Identity and Sibships

Identity analysis in *Colony2* detected seven individuals sampled twice each. Most were juveniles recaptured at the same site a year later, one adult was sampled at the same site 7 years apart (Appendix D: Table D1 in Supporting Information).

Sibship analysis in *Colony2* showed that 169 of 320 unique individuals (52.8%) were arranged in 28 families of two or more individuals, with the largest four families containing 16–30 full siblings (Table 3, Table D2 in Supporting Information). Full‐sibs from 18 families (including the three largest ones) were sampled from the same site, while those from 10 families were sampled from two sites (two from Sites 1–2, four from Sites 2–3, one from Sites 5–6, two from Sites 8–9, and one from Sites 10–12; Table 3). Ignoring individuals captured as adults, 12 families included individuals born in different cohorts: full‐sibs from six families were born in two consecutive years, from three families in three consecutive years, and from three families in two nonconsecutive years; Table 3. Some families also contained adults captured across different years. For example, Family 1 comprised adults captured at Site 5 in 2011, 2012, and 2014, in addition to juveniles captured in 2021 (confidently inferred to be born in 2019 and 2020), suggesting that a successful pair of adults bred together for multiple years in this site. Similarly, Family 2 sampled at Site 2 contained 18 juveniles captured as < 2YO, confidently inferred to be born in three consecutive years, indicating the same pair of fish bred for multiple years. Given the species' breeding biology, this result suggests limited numbers of breeders in some sites.

**TABLE 3:** Number of full‐siblings per family of two or more full‐sibs, inferred by *Colony2*, across sampling sites and inferred birth cohorts.

Family ID	Sampling site										*N*	Cohort of juveniles					Adult
	1	2	3	4	5	6	8	9	10	12		2018	2019	2020	2021	2022	
1					30						30		5	10			15
2		20									20	6	9	4			1
3		18									18			18			
4		5	11								16	2	2	12			
5	6	2									8		1	3	1		3
6				8							8			7			1
7					3	4					7		1			3	3
8		6									6	3	3				
9		2	4								6	1		5			
10		6									6	4	1				1
11		6									6			6			
12		1	5								6	1		5			
13	3	1									4		1				3
14		1	1								2	1	1				
15		2									2						2
16		2									2		2				
17		2									2	1	1				
18			2								2			2			
19							2				2			2			
20							2				2			2			
21							2				2			2			
22						2					2					2	
23							1	1			2					2	
24							1	1			2				1	1	
25							2				2					2	
26							2				2			2			
27					2						2			2			
28									1	1	2						2

*Note:* Shaded column (*N*‐ sample size) separates two ways the samples were tabulated: left‐ per sampling site, right‐ per cohort.

Sibship analysis revealed that two of three admixed individuals were full siblings (MP_M287 born 2021, captured in 2023 at Site 9 and MP_M320 born 2022, captured at Site 8 in 2023). The third admixed individual (MP_M325 born 2021, captured at Site 8 in 2023), was identified as their half‐sib. Therefore, one or two translocated Cataract Reservoir fish successfully bred in the Murrumbidgee, including one that bred with the same local partner for two consecutive years.

### 3.3 Barriers to Gene Flow and Dispersal

The geographic restriction of full siblings to one or two neighbouring sites (Table 3) and the evidence of genetic structure (Figure 3, Appendix C in Supporting Information) indicated barriers to gene flow between Sites 3–4, and 4–5, with only occasional connection between Sites 1–2, 5–6, and Site 6 and the more downstream breeding sites 7–9 (Table 3, Table D2 in Supporting Information). Only 9 individuals were sampled at Site 1 (sampled only in 2023, a year of recruitment failure), these belonged to Families 5 and 13, with three other family members captured at Site 2. The latter likely represent downstream dispersers from Site 1, with high flow years in 2021 and 2022 likely facilitating such movement (Table D2 in Supporting Information, Figure D1 in Supporting Information). Admixture plots (Figures C4 and C6 in Supporting Information) also indicated a migrant at Site 5 from one of the downstream sites: it clustered with individuals at Sites 7–9 on PCA (Figure 3), thus it is likely to be an upstream disperser from Sites 7–9 via Site 6 to Site 5. Some downstream migration from Site 5 to Site 6 was revealed by admixture analysis, confirmed by spread of Family 7 siblings across these sites.

Analysis of immigration rates in *BA3‐SNPs* showed that the overall migration was low, and mainly restricted to between neighbouring sites (Tables C2 and C3 in Supporting Information). While > 30% of individuals in Sites 6, 7, and 9 appear to be derived from migrants from upstream and/or downstream sites, less than 10% of individuals from Sites 5 and 8 are migrants, although these sites appear to be sources of migrants to other sites. Per‐site analysis showed some degree of downstream (from Site 1 to 2, from 5 to 6, and from 8 to 9) and upstream migration (from Site 3 to 2, from 4 to 3, and from 8 to 7 and 6).

Overall, our results of admixture, immigration rates, and spatial distribution of siblings challenge and clarify some of our prior hypotheses about Macquarie perch population connectivity in the Murrumbidgee (1|→2?→3|→4?→5←→6←→7?→8←→9|→10?→11?→12; symbols defined as in the legend of Figure 1). Downstream dispersal 1|→2 was supported by *BA3‐SNPs* analysis of sites (Table C2 in Supporting Information) but that of seven genetic groups (Table C3 in Supporting Information) suggests some upstream migration, which we denote as “←,” so occasional bidirectional dispersal 1←→2 can be inferred. Site 2?→3 connectivity was clarified by sibship and admixture (Figure C6 in Supporting Information), with prevalence of upstream 2←3 dispersal suggested by *BA3‐SNPs* (Table C2 in Supporting Information). In contrast to our expectation of mainly downstream dispersal 3|→4?→5, only upstream dispersal 3←4 was supported, with no gene exchange between 4 and 5 (4||5, where “||” indicates lack of gene flow in either direction; Tables C2 and C3 in Supporting Information). Bidirectional 5←→6 connection was supported (5→6: Table C2 in Supporting Information, 5←6: Figure C6 in Supporting Information), but presumed connection 6←→7 and downstream dispersal 7?→8 were not; instead no dispersal between 6 and 8 was found, and upstream dispersal 7←8 appears more likely (6||7|←8; Figure C5 in Supporting Information, Table C2 in Supporting Information). Regarding hypothesized 8←→9, supported by sibship distribution, only 8|→9 was supported by *BA3‐SNPs* (Table C2 in Supporting Information). The most downstream sites, 10–12, appeared isolated from the recruitment reach (Figures C4 and C6 in Supporting Information). Connection 10←→12 was apparent from sibship analysis (Table D2 in Supporting Information), although small sample sizes at these sites limits inference. Therefore, we could summarize our new understanding of connectivity as 1←→2←3←4||5←→6||7←8|→9||10←→12.

### 3.4 Heterozygosity and Inbreeding

For the Murrumbidgee individuals of all ancestries, PHt increased significantly with distance from the most upstream Site 1 (*p* < 0.001; Appendix E: Figure E2 in Supporting Information), and PHt averaged per site differed significantly among sites (ANOVA *p* < 0.001), generally increasing from upstream to downstream sites (Figure E3 in Supporting Information). The exception to this general trend was geographically intermediate Site 4 having very low heterozygosity and all eight individuals representing the site being members of a single full‐sib family.

PHt also differed significantly across cohorts (ANOVA *p* < 0.001; Figure E4 in Supporting Information), suggesting it was affected by environmental variation (such as flow) specific to year of birth. The cohort born in 2019 with the lowest water flow had the lowest mean heterozygosity (significantly lower than 2020, 2021, and 2022 cohorts; Tukey tests *p* < 0.01; Table E2 in Supporting Information), followed by 2018 (significantly lower than 2021 and 2022; *p* < 0.01); the 2020 cohort showed intermediate heterozygosity (significantly lower than 2021; *p* < 0.05), followed by 2022, and the highest heterozygosity was seen in the 2021 cohort (Table E1 in Supporting Information).

**FIGURE 4:**
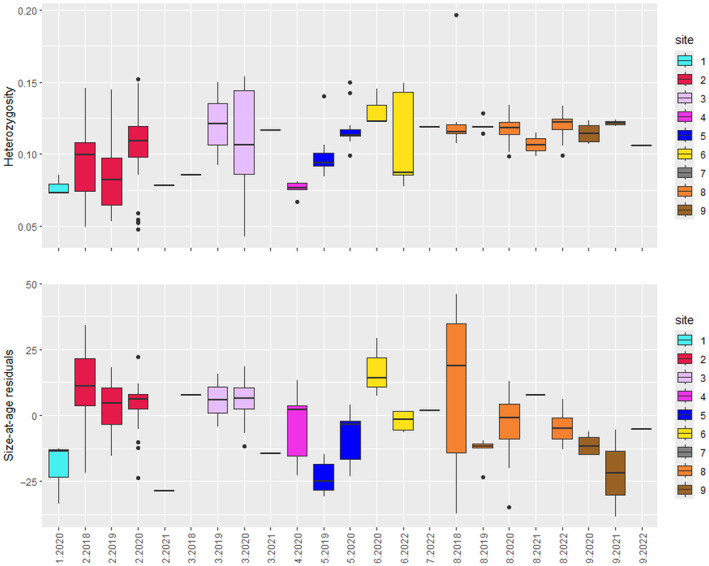
Individual heterozygosity, and size‐at‐age residuals per site for each cohort of juveniles.

Observed heterozygosity varied across cohorts within the same capture site (Figure 4, Figure E4 in Supporting Information). Of site‐by‐cohort groups of > 10 individuals, the lowest heterozygosity was in cohorts 2019 and 2018 born in drought years and captured in the upstream Site 2, followed by the 2020 cohort captured in Sites 2 and 3, heterozygosity was moderate in the 2020 cohort captured in Sites 5 and 8 and highest in the 2022 cohort captured in Site 8 (Table 4). Inclusion of the admixed individuals consistently resulted in higher estimates of observed heterozygosity (Table 4). Inbreeding in the Murrumbidgee, as calculated from population values of *H*e_Murrumbidgee_ = 0.128 and *H*e_CataractReservoir_ = 0.181 (Table E3 in Supporting Information) was *F*_e_ = 1—*H*e_Murrumbidgee_/*H*e_CataractReservoir_ = 0.293, that is, higher than the level that results from full‐sib or parent–offspring matings (0.25). Inbreeding was even stronger at individual sites (based on He values in Table 4, scaled by Cataract Reservoir), with *F*_e_ ranging from 0.298 at Site 6 and 0.304 at Sites 8 and 9 (both with admixed individuals), through 0.365 at Site 5, to 0.442 at Site 3 and 0.431 at Site 2, that is, generally increasing in the upstream direction.

**TABLE 4:** Observed (*H*o‐ mean, SD *H*o‐ standard deviation) and expected (*H*e‐ mean, SD *H*e‐ standard deviation) heterozygosity across the set of 3173 SNPs variable in individuals captured in the Murrumbidgee River, and *LDNe* estimates of effective population size (${\hat{N}}_{e}$ or ${\hat{N}}_{b}$—mean, CI low and CI high—low and high bounds of confidence intervals obtained by jackknife method) from the large dataset.

Categories/groups	Sample size	*H*o	SD *H*o	*H*e	SD *H*e	*F*_IS_	SD *F*_IS_	${\hat{N}}_{b}$ or ${\hat{N}}_{e}$	CI low	CI high
Site 2.2018	24	0.098	0.170	0.104	0.172	0.061	0.239	3	2	7
Site 2.2019	23	0.087	0.153	0.100	0.167	0.107	0.256	3	2	4
Site 2.2020	33	0.107	0.192	0.099	0.169	−0.031	0.216	12	9	17
Site 3.2020	33	0.112	0.197	0.101	0.171	−0.063	0.172	9	6	15
Site 5.2020	12	0.122	0.199	0.114	0.175	−0.018	0.246	17	9	54
Site 5.adults	18	0.105	0.172	0.112	0.173	0.091	0.273	12	3	63
Site 8.2020	47	0.121	0.181	0.122	0.178	0.015	0.146	128	110	173
Site 8.2022 no admixed	22	0.125	0.190	0.122	0.179	−0.003	0.199	99	55	239
Site 8.2022 with admixed	23	0.129	0.189	0.126	0.178	0.001	0.195	12	2	30,450
Site 8.adults	13	0.123	0.195	0.119	0.179	0.006	0.248	131	81	287
Site 9.adult	16	0.120	0.189	0.119	0.179	0.027	0.237	76	52	169
Pooled.sites2.3.2020	66	0.110	0.185	0.104	0.171	−0.023	0.152	6	4	8
Pooled.sites8.9.2020	51	0.121	0.180	0.122	0.178	0.017	0.145	86	70	106
Pooled.sites7.8.9.2022	24	0.124	0.188	0.122	0.178	−0.001	0.189	91	55	230
Pooled.sites7.8.9.adult	31	0.121	0.185	0.122	0.180	0.019	0.175	110	83	147
all.sites.2018	31	0.102	0.158	0.122	0.177	0.153	0.264	4	3	5
all.sites.2019	37	0.097	0.142	0.130	0.180	0.198	0.260	4	3	7
all.sites.2020	141	0.113	0.153	0.139	0.183	0.107	0.177	4	4	4
all.sites.2022 no admixed	29	0.122	0.178	0.129	0.181	0.058	0.215	18	9	47
all.sites.2022 with admixed	30	0.125	0.178	0.132	0.180	0.053	0.208	10	3	27
all.sites.adults	70	0.112	0.148	0.144	0.185	0.166	0.215	8	6	11
Site 2	87	0.099	0.164	0.103	0.170	0.031	0.142	4	3	6
Site 3	38	0.113	0.198	0.101	0.172	−0.062	0.160	11	7	17
Site 5	37	0.110	0.173	0.115	0.174	0.052	0.197	27	15	58
Site 6	12	0.122	0.189	0.127	0.183	0.061	0.282	5	2	11
Site 8 no admixed	96	0.122	0.180	0.123	0.179	0.012	0.113	112	98	132
Site 8 with admixed	98	0.124	0.179	0.126	0.178	0.012	0.112	49	19	244
Site 9 no admixed	23	0.120	0.185	0.121	0.179	0.028	0.211	95	64	136
Site 9 with admixed	24	0.124	0.183	0.126	0.178	0.029	0.202	8	2	1101
all.inds no admixed	315	0.111	0.146	0.142	0.184	0.104	0.174	4	4	6
all.inds with admixed	318	0.112	0.146	0.143	0.184	0.097	0.171	5	4	6

*Note:* Three sets of groups were assessed (each *N* > 10): Site‐per‐cohort, individuals born in the same year and at the same site (*site.cohort*); pooled sites per cohort, all individuals born in the same year regardless of the site (all.sites.*cohort*); pooled cohorts per site, all individuals captured at the same site regardless of year of birth (*site*); and all individuals (all.ind). Groups that included admixed individuals or a recaptured individual of Cataract Reservoir ancestry were analyzed with and without these individuals (the former are in red font).

For juveniles of Murrumbidgee‐only ancestry, sites alone explained 20.5% of the variance in heterozygosity (*p* < 0.001; adjusted *R* ^2^ = 0.205), indicating that population structure due to isolation was a major driver of genetic drift in parental populations (Appendix F: Table F1 in Supporting Information). Cohorts alone explained 8.3% of variance (*p* < 0.001; adjusted *R* ^2^ = 0.083; Table F1 in Supporting Information). Including both site and cohort as fixed effects significantly improved the model fit, although only site terms were significant (Table F1 in Supporting Information). Addition of site‐by‐cohort interaction did not significantly improve the model.

Individual heterozygosity (PHt) calculated from all individuals and averaged across ancestries was 2× higher in fish of admixed ancestry compared to individuals of Murrumbidgee ancestry and 1.3× higher than fish of Cataract Reservoir ancestry (ANOVA *p* < 0.001; all pairwise post hoc Tukey tests *p* < 0.005; Table E1 in Supporting Information, Figure E1 in Supporting Information). Cataract Reservoir individuals were 1.6 times more genetically diverse than Murrumbidgee ones. This indicated that the addition of Cataract Reservoir individuals to the Murrumbidgee population will add novel alleles.

### 3.5 Effective Population Sizes

Site‐by‐cohort *LDNe* analyses of the complete SNP dataset yielded the largest estimates of numbers of breeders ${\hat{N}}_{b}$, and per‐generation effective population sizes *N*_e_ (for adults collected across years) for the lower reach Site 8 (${\hat{N}}_{b}$ = 128 in 2020, 99 in 2022; ${\hat{N}}_{e}$ = 131; Table 4), followed by *N*_e_ for the next downstream Site 9 (${\hat{N}}_{e}$ = 76). The lowest number of breeders was at the most upstream analyzed Site 2 (${\hat{N}}_{b}$ = 3 in 2018 and 2019, 12 in 2020), followed by Sites 3 (${\hat{N}}_{b}$ = 9 in 2020) and 5 (${\hat{N}}_{b}$ = 17 in 2020; ${\hat{N}}_{e}$ = 12). Consistent with predictions, at sentinel Site 2 cohorts born in low‐flow years 2018 and 2019 appear to have had many fewer parents than the cohort born in 2020, which suggests that low flow might preclude movement of adults to sites upstream for spawning, or that local spawning sites are of lower availability (riffles too shallow to access/spawn in) or poorer quality (sediment on spawning sites or eggs) in worse drought years.

Correction for the biases due to iteroparity, age structure and overlapping generations (as ${\hat{N}}_{b}$/0.887), yielded *N*_b__adj_ estimates—numbers of breeders per reproductive cycle—of 3–14 for Site 2, 10 for Site 3, 19 for Site 5, and 112–144 for Site 8, suggesting levels of inbreeding such that inbreeding depression is likely at most sites.

Pooling low‐differentiation sites within cohorts resulted in lower values of ${\hat{N}}_{b}$ compared to the per‐cohort value for one or more individual sites (Table 4). Pooling individuals across sites, some of which were disconnected (Appendix C in Supporting Information), resulted in extremely small estimated *N*_e_ from the large SNP dataset. Per‐cohort analyses of individuals from all sites pooled (i.e., completely ignoring population structure) yielded much smaller estimates of the number of breeders (${\hat{N}}_{b}$ = 4 for cohorts 2018, 2019, and 2020, and 18 for cohort 2022; ${\hat{N}}_{e}$ = 8; Table 4). Pooling individuals that were captured at the same site but born in different years (i.e., ignoring cohorts) yielded *N*_e_ estimates that were lower compared to per‐site per‐cohort analyses, but higher compared to all sites analyses. Pooling all individuals of Murrumbidgee‐only ancestry resulted in ${\hat{N}}_{e}$ = 4. Including admixed individuals resulted in *N*_e_ estimates that were a fraction of those from analyses that excluded these individuals, despite their small number/proportion (site 8.2022 no admixed: 99, with admixed: 12; all.sites.2022 no admixed: 18, with admixed: 10; site 8 no admixed: 112, with admixed: 49; site 9 no admixed: 95, with admixed: 8; Table 4).

### 3.6 Models of Juvenile Growth and Inbreeding Depression

Population structure and environmental conditions during birth‐years appear to influence growth rate of Murrumbidgee‐only ancestry juveniles. Sites alone explained 23.3% of variance in size‐at‐age residuals (*p* < 0.001; adjusted *R* ^2^ = 0.233; Appendix G: Table G1 in Supporting Information). Growth rate differed non‐linearly with distance from the most upstream site (Table G2 in Supporting Information, Figure G1 in Supporting Information). Cohort alone explained 11.6% of the variance in size‐at‐age residuals (*p* < 0.001; adjusted *R* ^2^ = 0.116; Table G1 in Supporting Information). Cohort 2018 had significantly larger mean size‐at‐age residuals than did cohorts 2019–2022 (post hoc Tukey tests *p* < 0.001; Table G3 in Supporting Information, Figure G2 in Supporting Information), mirroring the result for the nearby Cotter River where the 2018 cohort may have experienced similar weather conditions (Pavlova et al. 2024). Including both site and cohort as fixed effects (*p* < 0.001; adjusted *R* ^2^ = 0.297) significantly improved the fit of single‐predictor models (*p* < 0.001) with most of the terms of being significant (Table G1 in Supporting Information). Including the interaction between site and cohort (*p* < 0.001; adjusted *R* ^2^ = 0.352; Table G1 in Supporting Information) further improved the model (*p* = 0.001), indicating that the effect of environmental conditions on growth may differ among sites (Figure 4). However, including heterozygosity in addition to site, cohort and their interaction as fixed effects (*p* < 0.001; adjusted *R* ^2^ = 0.353; Table G1 in Supporting Information) did not improve the model fit (*p* > 0.05), suggesting habitat quality moderated by environmental conditions as major drivers of differences in juvenile growth rate.

## 4 Discussion

Macquarie perch is a good example of a threatened species where anthropogenic habitat fragmentation disrupted between‐population (Pavlova et al. 2017) and within‐population connectivity (this study), resulting in many small, isolated, genetically depauperate, and highly inbred isolated remnants. Lack of connectivity among the upper reach sites of the Murrumbidgee Macquarie perch population, very strong inbreeding, low genetic diversity, and low *N*_e_ within disconnected habitat fragments highlight the need for urgent actions to reconnect these fragments. Our results strongly support previous inferences—based on limited samples—of reduced genetic diversity and high inbreeding for this population (Farrington, Lintermans, and Ebner 2014; Pavlova et al. 2017). Our estimate of ${\hat{N}}_{e}$ = 12 for Site 5 was lower than earlier microsatellite‐based estimates for the same site done using the same method: ${\hat{N}}_{e}$ = 23 from fish captured in 2011–2012 (Pavlova et al. 2017) or ${\hat{N}}_{e}$ = 64 from fish captured prior to 2007 (Farrington, Lintermans, and Ebner 2014), suggesting a possible decline in the number of breeders over a few generations. But it is also possible that patterns of isolation are temporally dynamic, with higher immigration in higher flow years leading to lower ${\hat{N}}_{e}$ due to elevated mixture LD introduced by migrants from genetically differentiated sites (Waples 2024b).

Effective population size *N*_e_ is an important parameter to monitor during conservation interventions, because it can flag inbreeding relevant to population genetic health (Hoban, Paz‐Vinas, et al. 2021). However, we showed that assessment of per‐generation *N*_e_ and single‐year *N*_b_ estimated using LD approaches may be biased if population substructure is not accounted for, leading to lower estimates from a pool of individuals from differentiated subpopulations. This supports earlier findings that a metapopulation comprising strongly differentiated demes has a dramatically smaller estimate of meta *N*_e_ compared to the sum of the local *N*_e_ estimates (Palstra and Ruzzante 2011; Baalsrud et al. 2014; Waples 2024b). It also highlights the importance of identifying the appropriate scale on which distinct demographic and genetic processes operate. Given limited connectivity across several river fragments, the Murrumbidgee Macquarie perch metapopulation could approximately conform to Wright's island model. Assuming that our sampling regime adequately covers the breeding neighborhoods within each fragment (Neel et al. 2013), and gene flow is low, each local ${\hat{N}}_{e}$ estimate should reflect the per‐generation *N*_e_ of each fragment, and the total per‐generation *N*_e_ in the upper Murrumbidgee Macquarie perch population (relevant to the *N*_e_ < 1000 criterion for reduced adaptive potential) should be larger than each local ${\hat{N}}_{e}$, but smaller than the sum of local ${\hat{N}}_{e}$ estimates for each genetically‐isolated river fragment (Waples 2024a). In contrast, for a system with high gene flow (> 10%), the level of inbreeding (and *N*_e_) will be the same in all connected sub‐populations, and thus the local *N*_e_ estimate will reflect metapopulation‐ rather than local *N*_e_ (Ryman, Laikre, and Hössjer 2023; Waples 2024b). Total *N*_e_ of a metapopulation created by habitat fragmentation of a formerly connected population could be up to two orders of magnitude lower than the maximum total number of individuals in the system (Gilpin 2008). When strong population structure is present and gene flow is low, accounting for population genetic structure is essential for delivering meaningful genetic estimates of local *N*_e_. While pooling individuals from sampling sites connected by gene flow provides meaningful population‐wide estimates of *N*_e_ (Waples 2024b), large‐scale inferences from pooled disconnected sites may need to be reevaluated.

Because genetically divergent migrants introduce mixture LD, which lowers the population *N*_e_ estimate (Waples 2024b), LD‐based estimates of *N*_e_ are not suitable for monitoring progress of augmented gene flow and genetic rescue. In our data, addition of genetically differentiated migrants or admixed offspring decreased the population *N*_e_ estimate, as was also seen in another population (Pavlova et al. 2024). Instead, heterozygosity, which is significantly higher in the admixed individuals here (and which will usually apply in other systems) and elevated the overall population genetic diversity estimates, appears to be a suitable metric for monitoring. SNP‐based heterozygosity, as assessed here, might be appropriate only for within‐population comparisons within a single study (Schmidt et al. 2021). For long‐term monitoring or multi‐population studies, unbiased heterozygosity, which accounts for invariable and multi‐allelic sites that are filtered from SNP datasets (Schmidt et al. 2021; Sopniewski and Catullo 2024), should be used to enable comparisons between studies.

Because loss of connectivity due to fragmentation poses one of the greatest threats to freshwater biodiversity (Liermann et al. 2012; Crook et al. 2015; Gido et al. 2016), threatened species managers would benefit from embracing regular assessment of population genetic structure. This will highlight management priorities such as the need to increase connectivity, employ within‐population genetic augmentation and/or introduce new genetic sources (Pierson et al. 2016; Frankham et al. 2017; Taylor, Dussex, and Van Heezik 2017). The development of genetic structure at small spatial scales in response to novel barriers to dispersal, as observed among the Murrumbidgee sites for Macquarie perch, depends on the time since barriers were established, effective population size, levels of gene flow, and the spatial scale of organismal dispersal (Landguth et al. 2010; Coleman et al. 2018). While many threats have contributed to the demise of Macquarie perch across its range (Commonwealth of Australia 2018; Lintermans et al. 2019), the genetic structure, loss of diversity through drift, and strong inbreeding of the upper Murrumbidgee population is almost certainly the result of a loss of riverine connectivity within the population due to permanent diversion of > 90% of river flows away from the Murrumbidgee. While occasional higher‐flow years may facilitate some dispersal between adjacent or nearby sites, currently there is no way to augment the poor genetic diversity of the isolated sites other than through human‐assisted translocations. Understanding connectivity helps decision‐making by providing better understanding of which locations are isolated and need rescue, which might be good sources of migrants to other sites and rivers, and what migration is likely to occur with and without direct intervention. For example, sites with a high proportion of non‐immigrants and a high contribution to other sites might be important sources of migrants to other sites in the river (and to other rivers), whereas sites with low proportions of non‐immigrants might be migration sinks. Our understanding of connectivity in the Murrumbidgee is still incomplete because several sites had only 1 or 2 years of sampling, and so the influence of flow variability between years on connectivity is not well understood.

The low genetic diversity and high inbreeding in the upper Murrumbidgee population of Macquarie perch is more severe than that of the Cotter River, a nearby tributary of the Murrumbidgee. The Cotter may benefit from proximity to a large reservoir refugium at its downstream end (Ebner, Lintermans, and Dunford 2011). Such reservoirs provide more stable habitats and environmental conditions than adjacent rivers. They also facilitate retention of adults in years of high water temperatures or low flow, and rapid recolonization of upstream river segments when conditions are more hospitable (Pavlova et al. 2024). Unlike in the Cotter River, where low environmental flows from an upstream water supply reservoir limit connectivity in some years, in the upper Murrumbidgee the diversion of natural flows has resulted in previously ephemeral natural barriers to upstream fish movement becoming largely permanent. Prior to Tantangara Dam's construction, cascades in the deeply incised, bedrock‐confined, rocky gorge‐dominated upper Murrumbidgee River would have been regularly inundated by winter/spring floods and high annual baseflows between July and October (Pendlebury et al. 1997). Tantangara Dam has resulted in a fourfold reduction of large downstream flows and frequency of large flow events, and significantly reduced base flows in all seasons (Snowy Scientific Committee 2010). Small populations above instream barriers are at higher risk of extinction (Whiteley et al. 2013). The existing environmental flow releases in the Murrumbidgee are inadequate for supporting Macquarie perch (Snowy Scientific Committee 2010). Improving and restoring connectivity between upper, middle and lower sites within the recruitment reach is essential for the persistence of this threatened species, through being a key natural population. Ideally, improved connectivity would be achieved through the provision of adequate environmental flow releases from Tantangara Dam, which would provide regular natural connectivity among sites through fish dispersal. Such releases could assist in inundating barriers to Macquarie perch movement, and also promote instream recovery of a range of aquatic taxa such as other fish species, threatened freshwater crayfish and other aquatic vertebrates and invertebrates by reducing the accumulation of instream sediments, restoring substrate diversity and function (e.g., spawning and interstitial shelter), and deceasing riparian encroachment and channel contraction (Pendlebury et al. 1997; Lintermans 2023b). To inform river flow management and to increase connectivity and genetic recovery among Macquarie perch sites, a better understanding is needed of the environmental conditions (e.g., season, flow magnitude) that enhance or support fish movements, including frequency and magnitude (distance and number of individuals) of downstream dispersal under favorable conditions, and flow magnitudes that submerge instream barriers of a range of sizes to facilitate upstream fish migration (Lintermans 2023c).

If achieving regular adequate flows is currently challenging due to competing interests for water availability or limited capacity of outlet structure to release the flows required to ameliorate barriers, genetic rescue by exchange of individuals among the river sites should be implemented immediately and regularly. Such augmented gene flow would increase the extremely low *N*_b_ and genetic diversity at all (especially upstream) Murrumbidgee sites, decrease inbreeding, and reduce genetic drift. Individuals should be moved in upstream and downstream directions (including sourcing fish in the most downstream sites outside of the recruitment reach where possible), aiming to reduce within‐Murrumbidgee population structure and achieve considerable admixture among fish born at different Murrumbidgee sites. Given the small number of breeding adults at each site, removal of adults may significantly reduce spawning success at each site. To avoid detrimental effects of removal of adults from their local sites, 1–2YO individuals or subadults approaching maturity (2–3YO) could be used for translocations among sites—these are expected to have higher survivorship than 0–1YO individuals (Todd and Lintermans 2015). Adhering to principles of adaptive management, genetic monitoring should continue, with success measured by reduction of population genetic structure and increase in genetic diversity of individuals born in the Murrumbidgee. Even if complete reconnection of sites within the upper Murrumbidgee is achieved, the total genetic diversity of this population will be lower than that of the Cataract Reservoir population, which originated from the lower Murrumbidgee River ~100 years ago. This supports the urgent need to further augment the Murrumbidgee population with fish from other populations. Sourcing individuals from other populations should help to increase genetic diversity of the local population faster, and to a greater extent for the same number of migrants (Frankham et al. 2019; Nistelberger et al. 2024).

This study adds to a growing number of genetically monitored cases of genetic rescue, informing design of interventions for threatened species (Weeks et al. 2017; Poirier et al. 2019; Fitzpatrick et al. 2020). In the short period since genetic augmentation commenced in the middle and downstream sites in the upper Murrumbidgee, 1.65% (three of 182) of Macquarie perch born after translocations from Cataract Reservoir commenced were of mixed ancestry, and 0.7% (one of 139) translocated fish bred. There were at least two cases of a translocated fish breeding with a local one, producing individuals that were more genetically diverse than any fish of either parental population. For a group‐spawning species such as Macquarie perch, detection of repeated breeding with the same partner may indicate strong mate‐limitation. The detection of breeding at downstream Sites 8 and 9 in 2021 means that only the release in 2020 of 41 Cataract fish in Sites 7 and 8 resulted in the detection of successful interbreeding (the following year). The release in 2022 of 98 Cataract fish to Sites 5, 7, and 8 did not result in detected interbreeding, although Site 5 was not monitored in 2023, limiting our ability to detect interbreeding. These results are somewhat similar to the modest early success of genetic augmentation in the nearby Cotter River, where only 0.45% of juveniles were of mixed ancestry, and only 1.4% of translocated fish (a single Cataract individual) had produced offspring within 3 years of augmentation (Pavlova et al. 2024). This limited success could reflect periods of adjustment for individuals of this long‐lived species to the environmental and social conditions of the recipient site, and attainment of larger body size (Pavlova et al. 2024). While both studies document only modest early success of genetic augmentation, translocation of many more individuals and comprehensive follow‐up monitoring is needed. The actual number of individuals to be moved will depend on the conservation management goal. For example, if the goal is to lower the inbreeding in each site to *F*_e_ < 0.1, that is, half the level above which severe harmful inbreeding depression is usually observed (Frankham 1995), then the proportion of adult breeders derived from outbred migrants can be calculated for each site as *f*_migrant_ = 1 − sqrt(*F*_e __augmented_/*F*_e __inbred_site_) (equation 12.2 of Frankham et al. 2017). For four sites with averaged adjusted per‐cohort *N*_b_ estimates, this proportion will be 0.45 for site 8, 0.49 for site 5 and 0.53 for Sites 2 and 3. The number of effective migrants per site *N*m then can be estimated as *N*m = *f*_migrant_ × *N*_b_/(1 − *f*_migrant_) (detailed explanation of calculations in Pavlova et al. 2024), which will be 61 for Site 8, 14 for Site 5, and 3 for Sites 2 and 3. However, what proportion of translocated fish will eventually become breeders is unknown, thus adaptive management, where the population is monitored for reduced population structure within the upper Murrumbidgee, reduced per‐site inbreeding, and increased overall population genetic diversity, represents a positive approach. Importantly, once inbreeding is substantially reduced, translocations will need to continue, albeit at a lower level, to ensure that inbreeding does not reappear in the future, and the population continues to receive variation to enable future adaptation (Ralls et al. 2020). Genetic augmentation and associated monitoring need to occur across all riverine fragments, that is, the entire recruitment reach of the upper Murrumbidgee. Given that multiple donor sources can provide greater success after translocation compared to a single‐source (Lutz et al. 2021; Nistelberger et al. 2024), sourcing migrants from more genetically diverse populations, including from other catchments (e.g., Yarra, Ovens, and Abercrombie Rivers) should be considered, because this could provide additional genetic variation fueling adaptation to changing environments. Given earlier recommendations of including riverine genetic stocks for genetic augmentation to support adaptation (or prevent maladaptation) to riverine conditions (Lutz et al. 2021), it is important to consider connectivity among the riverine fragments of source populations. If a fragmented population such as the Murrumbidgee is used as a source for genetic augmentation of other populations, individuals should be collected across multiple sites to avoid translocating close relatives to the same location, and to ameliorate the impact of somewhat inbred individuals by ensuring that they contain different genetic variation from each other, which will mix in future generations.

In the upper Murrumbidgee Macquarie perch, heterozygosity and growth rate were strongly affected by site and cohort, suggesting spatial and temporal environmental effects on both, but growth rate did not correlate with the level of individual inbreeding. Lack of detectable inbreeding depression contrasts to the findings for the nearby Cotter River Macquarie perch population, where the 2018 cohort (which was also born and lived its first year in stressful conditions of the lowest river flow), displayed a significant positive relationship between heterozygosity and growth, suggesting possible inbreeding depression (Pavlova et al. 2024). It is thus likely that in the upper Murrumbidgee, the heterogeneous environments of isolated fragments and flow conditions have had stronger effects on growth than inbreeding (Schons, Vitt, and Thünken 2022), or size‐at‐age residuals provide insufficient estimates of fitness. Interestingly, as in the Cotter, the highest growth was observed in low‐flow cohorts, possibly due to stronger selection for faster growth and maturation under stressful conditions (Pavlova et al. 2024), or the reallocation of energy from swimming to growth.

## 5 Management Recommendations

Providing best‐practice management to the spatially large but genetically compromised upper Murrumbidgee Macquarie perch population is essential to ensure that it persists, that its unique genetic diversity is not lost through genetic drift, and that the overall recovery of this threatened species is promoted through gene exchange among the major catchments of the Murray–Darling Basin. Four key actions are strongly recommended. Of highest priority is improving connectivity among sites within the upper Murrumbidgee River, by providing adequate flow releases from Tantangara Reservoir and human‐mediated exchange of migrants among four genetic groups (Sites 1–3, Site 4, Site 5, and Sites 7–9). Continuing wild‐to‐wild translocations from other populations of the Murray–Darling Basin is also of highest priority. These actions are expected to reduce inbreeding and genetic drift and increase genetic diversity and hence fitness and evolutionary potential. A genetic risk assessment for the species suggests it is highly unlikely that negative consequences of such admixture would occur (Pavlova et al. 2017). Stocking with genetically diverse (admixed) offspring should also be seriously considered as method to accelerate genetic rescue. Finally, genetic monitoring of Macquarie perch cohorts in the Murrumbidgee should be used to continue to evaluate the impact of interventions and adjust accordingly (see detailed recommendations in Appendix H in Supporting Information). If the upper Murrumbidgee River population is used as a source of individuals for genetic augmentation of other populations (e.g., the Cotter River, Pavlova et al. 2024), it should be included as one of two or more sources, to increase/maximize genetic diversity. Any individuals harvested as migrants should be replaced by migrants from other locations, and effects on source populations of harvesting for translocation should be monitored to ensure the number of breeders does not decline as a result (Mitchell et al. 2022).

## 6 Conclusions

The upper Murrumbidgee River Macquarie perch population, while having a spatially large distribution, is highly fragmented with low genetic diversity and low *N*_e_ estimates at many sites. Simply monitoring the number of 0–1 YO fish produced annually has masked the need for genetic rescue of the whole population as well as individual sites. Assessments of per‐generation *N*_e_ and single‐year *N*_b_ using LD approaches require accounting for population structure. Large‐scale inferences from pooled disconnected sites may need to be reevaluated. LD‐based estimates of *N*_e_ are not suitable for monitoring the progress of augmented gene flow. In contrast, heterozygosity, which increased in the Murrumbidgee River with the addition of migrants or admixed offspring, appears to be an adequate index for monitoring success of genetic augmentation. Threatened species conservation and recovery programs should embed the need for regular (3–5 yearly) reviews of critical conservation genetic parameters (genetic diversity, population structure, inbreeding, and *N*_e_) to inform evaluation of management priorities and results.

## Conflicts of Interest

The authors declare no conflicts of interest.

## Supporting information


Appendix S1.

## Data Availability

All data and R scripts for analyses used in this study will be available on Bridges data repository at https://doi.org/10.26180/25467973. Benefits from this research accrue from the sharing of our data and results on public databases as described above. Genotyping data and R script used for genetic analyses are also available in Dryad at https://doi.org/10.5061/dryad.j3tx95xqc.
