# Supplementary material for: Immediate Genetic Augmentation and Enhanced Habitat Connectivity Are Required to Secure the Future of an Iconic Endangered Freshwater Fish Population
Source: Evol Appl. 2024 Oct 12;17(10):e70019. doi: 10.1111/eva.70019 (PMC11470195; doi:10.1111/eva.70019)
Supplement: Supplementary file 1 — Appendix S1. [file EVA-17-e70019-s001.docx]

**Appendices for the manuscript:**

Pavlova, A., Pearce, L., Sturgiss, F., Lake, E., Sunnucks, P., & Lintermans, M. 2024. **Immediate genetic augmentation and enhanced habitat connectivity are required to secure the future of an iconic endangered freshwater fish population.** *Evolutionary Applications* doi: 10.1111/eva.70019.

All ***R scripts*** and ***data files*** used for the analyses are available in Bridges data repository at DOI <https://doi.org/10.26180/25467973>

File ***Murrumbidgee_Cataract.genetics.finess.csv*** contains field data and genetic results.

**Contents:**

[**Appendix A.** Per year summary of the Murrumbidgee River flow, the number and release location of fish translocated from Cataract Reservoir and recruitment success as assessed by the surveys (Table A1), and genetic samples used for this study (Table A2). 1](#_Toc174111141)

[**Appendix B.** Age and growth rate analysis for Murrumbidgee Macquarie perch. 2](#_Toc174111142)

[**Appendix C.** Additional filtering and additional analyses of population genetic structure 7](#_Toc174111143)

[**Appendix D.** Identity and sibship analyses in *Colony2*. 14](#_Toc174111144)

[**Appendix E.** Estimates of genetic diversity and effective number of breeders for various groups 17](#_Toc174111145)

[**Appendix F.** Results of linear models (LM) modelling Murrumbidgee Macquarie perch heterozygosity as a function of site and cohort for juveniles born 2018-onwards. Plot is on Fig. 4 of the main text 23](#_Toc174111146)

[**Appendix G.** Results of linear models (LM) of Murrumbidgee Macquarie perch growth residuals as a function of site, cohort and heterozygosity for juveniles born 2018-onwards (Table G1), posthoc Tukey tests for models LM.G.site and LM.G.cohort (Tables G2-G3) and plots of growth residuals by sites (Fig. G1), cohorts (Fig. G2) and heterozygosity (Fig. G3). 25](#_Toc174111147)

[**Appendix H.** Detailed recommendations for genetic management of Murrumbidgee Macquarie perch population. 31](#_Toc174111148)

# **Appendix A.** Per year summary of the Murrumbidgee River flow, the number and release location of fish translocated from Cataract Reservoir and recruitment success as assessed by the surveys (Table A1), and genetic samples used for this study (Table A2).

**Table A1.** Murrumbidgee Mean daily flow (ML/day) for the calendar years 2017 to 2023 (up to 8/Dec/2023), summary of translocations from Cataract Reservoir, and summary of recruitment success, measured for each cohort as a number of 0-1YO fish captured per net-night, as assessed by standard fyke net monitoring. Mean daily flow measured at a flow gauging stations at Yaouk, 8.25 km above site 1 and at site 7. The measurements at the two stations are highly correlated (R^2^=0.99; P<0.001).

| **Calendar Year** | **Murrumbidgee**  **mean daily flow (ML/day)** | | **Macquarie perch**  **translocations**  **from Cataract Reservoir** | | **Macquarie perch**  **recruitment success,**  **assessed by surveys** | | |
| --- | --- | --- | --- | --- | --- | --- | --- |
|  | **Yaouk** | **Site 7** | **N fish** | **Release sites** | **Cohort** | **Site 2** | **Site 8** |
| 2017 | 185.4 | 229.1 |  |  |  | NS | NS |
| 2018 | 90.6 | 86.1 |  |  |  | NS | NS |
| 2019 | 73.7 | 70.7 |  |  | 2018 | NS | 12 |
| 2020 | 234.0 | 301.8 | 41 | Sites 7, 8 | 2019 | 6 | 14 |
| 2021 | 425.4 | 662.1 |  |  | 2020 | 66 | 55 |
| 2022 | 678.1 | 1218.3 | 98 | Sites 5, 7, 8 | 2021 | 1 | 20 |
| 2023 | 232.6 | 309.2 |  |  | 2022 | 0 | 31 |

**Table A2.** Genetic samples genotyped for this study, tallied per *sampling* year per site. For analyses, sampling locations <2 km apart were combined. Cataract Reservoir individuals analyzed in this study were not the individuals translocated to Murrumbidgee.

|  | **Sampling year** | | | | | | | | | | | | | |  |
| --- | --- | --- | --- | --- | --- | --- | --- | --- | --- | --- | --- | --- | --- | --- | --- |
| **Waterbody/**  **Site** | **2002** | **2003** | **2007** | **2010** | **2011** | **2012** | **2014** | **2017** | **2018** | **2019** | **2020** | **2021** | **2022** | **2023** | **Total** |
| **Cataract Reservoir** |  |  |  |  |  |  |  | **27** | **28** | **12** |  |  |  |  | **67** |
| **Murrumbidgee R** | **1** | **1** | **1** | **1** | **2** | **1** | **22** | **0** | **0** | **2** | **43** | **144** | **36** | **76** | **330** |
| Site 1 |  |  |  |  |  |  |  |  |  |  |  |  |  | 9 | 9 |
| Site 2 |  |  |  |  |  |  |  |  |  |  | 29 | 48 | 9 | 7 | 93 |
| Site 3 |  |  |  |  |  |  |  |  |  |  |  | 31 | 7 |  | 38 |
| Site 4 |  |  |  |  |  |  |  |  |  |  |  |  |  | 8 | 8 |
| Site 5 |  |  |  |  | 2 | 1 | 15 |  |  |  |  | 19 |  |  | 37 |
| Site 6 |  |  |  |  |  |  |  |  |  |  |  |  |  | 12 | 12 |
| Site 7 |  |  |  |  |  |  |  |  |  |  |  |  |  | 3 | 3 |
| Site 8 | 1 | 1 |  |  |  |  |  |  |  |  | 14 | 37 | 18 | 30 | 101 |
| Site 9 |  |  |  |  |  |  | 7 |  |  |  |  | 9 | 2 | 7 | 25 |
| Site 10 |  |  |  | 1 |  |  |  |  |  |  |  |  |  |  | 1 |
| Site 11 |  |  | 1 |  |  |  |  |  |  |  |  |  |  |  | 1 |
| Site12 |  |  |  |  |  |  |  |  |  | 2 |  |  |  |  | 2 |
| **Total** | **1** | **1** | **1** | **1** | **2** | **1** | **22** | **27** | **28** | **14** | **43** | **144** | **36** | **76** | **397** |

# **Appendix B.** Age and growth rate analysis for Murrumbidgee Macquarie perch.

*Assigning cohorts*

**Figure B1.** Distribution of lengths for all 324 Murrumbidgee individuals with length data. The first cohort (length <100 mm) corresponds to individuals 0-1Year old (YO). Examination of length distributions for each sampling year (Fig. B2) revealed consistent breaks for 2021, 2022 and 2023, for which we assigned individuals 100-180 mm to 1-2YO, individuals 181-270 mm to 2-3YO, and individuals >271 mm to >3YO. For 2020, individuals 100-210 mm formed a distinct peak (Fig. B2) and were assigned to 1-2YO; 211-270 mm to 2-3YO, and criteria for >3YO were as above.


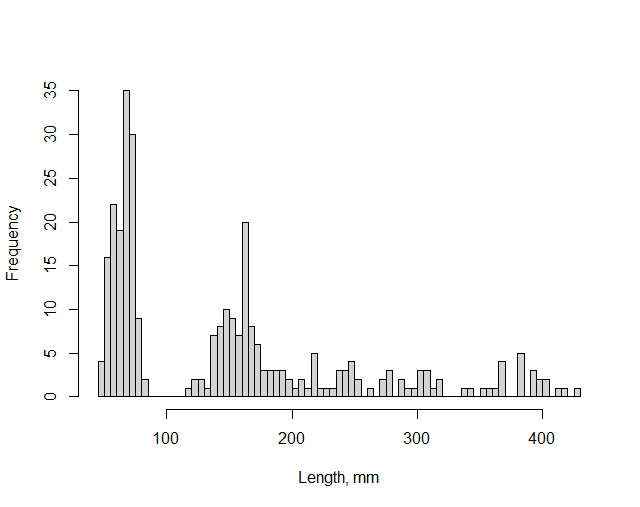


**Figure B2.** Length (in mm) distribution of Murrumbidgee Macquarie perch per sampling year.

**
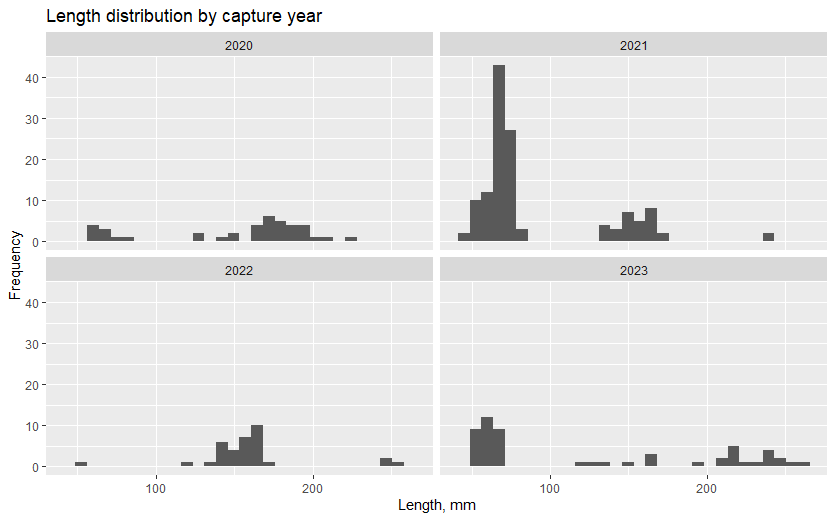
**

**Figure B3.** Distribution of Murrumbidgee Macquarie perch lengths (in mm) coloured by assigned age categories. Three individuals <250 mm in “adult” category are those sampled in 2007- 2011.


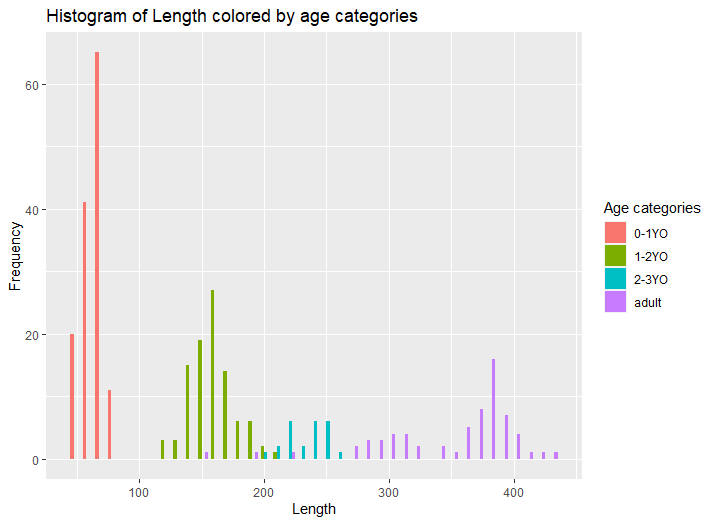


**Table B1** Sample sizes of Murrumbidgee Macquarie perch juvenile (<3YO) age categories per cohort (Fig. B3)

| **Cohort** | **0-1YO** | **1-2YO** | **2-3YO** | **Sample size** |
| --- | --- | --- | --- | --- |
| 2017 |  |  | 1 | 1 |
| 2018 |  | 30 | 2 | 32 |
| 2019 | 9 | 29 | 3 | 41 |
| 2020 | 97 | 30 | 18 | 145 |
| 2021 | 1 | 7 |  | 8 |
| 2022 | 30 |  |  | 30 |
| **Sample size** | **137** | **96** | **24** | **257** |

**Figure B4.** Distribution of assigned age groups among Murrumbidgee cohorts (x-axis)


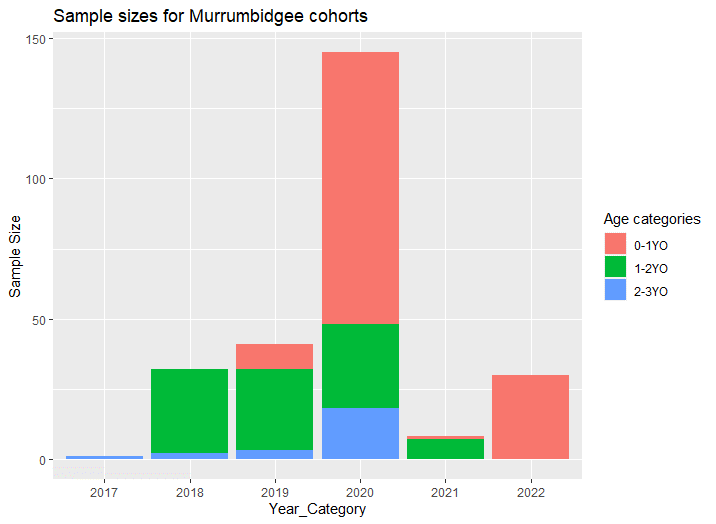


Cohort

*Gompertz Growth model*

For 257 Murrumbidgee Macquarie perch juveniles aged as 0-1YO, 1-2YO or 2-3YO growth residuals were estimating by fitting Gompertz Growth model. Initial parameters used were b1 = 200, b2 = 0.5, b3 = 0.3. Results are below:

Formula: Length ~ b1 * exp(-b2 * exp(b3 * inf.age.at.capture))

Parameters:

Estimate Std. Error t value Pr(>|t|)

b1 320.99583 14.95307 21.47 <2e-16 ***

b2 2.10878 0.03722 56.66 <2e-16 ***

b3 -0.81503 0.04770 -17.09 <2e-16 ***

---

Signif. codes: 0 ‘***’ 0.001 ‘**’ 0.01 ‘*’ 0.05 ‘.’ 0.1 ‘ ’ 1

Residual standard error: 13.02 on 254 degrees of freedom

Algorithm "port", convergence message: relative convergence (4)

**Figure B5**. Distribution of Murrumbidgee Macquarie perch lengths (in mm) per inferred age at capture (all sampling years).


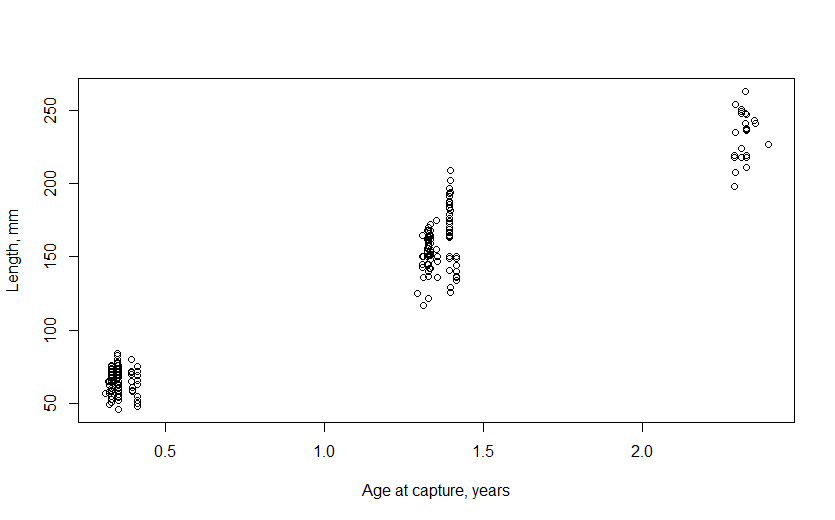


**Figure B6.** Growth residuals of Murrumbidgee Macquarie perch from the Gompertz growth model, vs inferred age at capture (all sampling years).


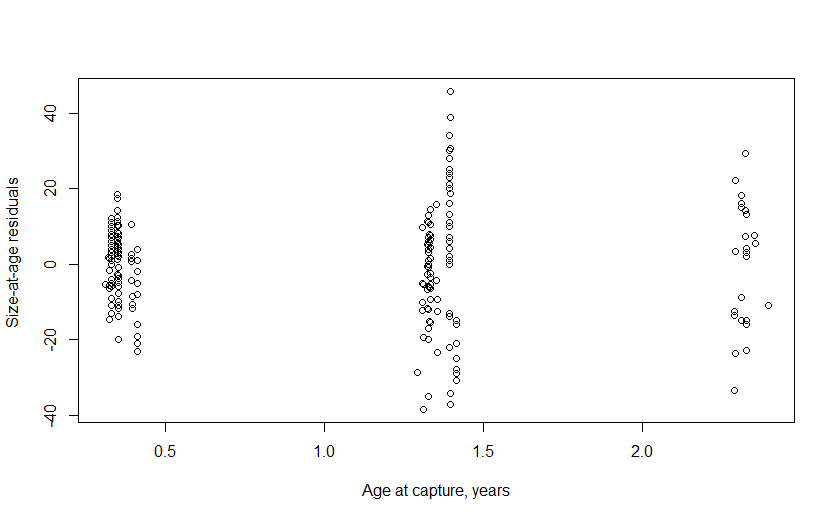


**Figure B7.** Distribution of growth residuals of Murrumbidgee Macquarie perch from the Gompertz growth model


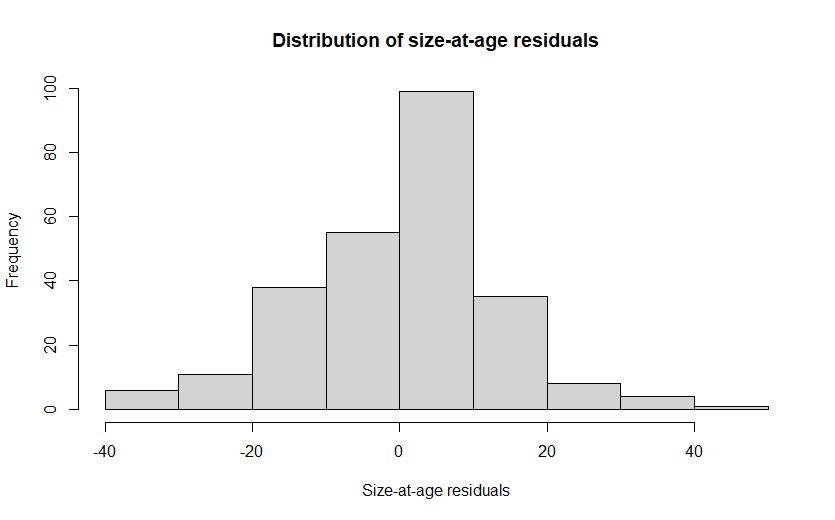


# **Appendix C.** Additional filtering and additional analyses of population genetic structure

To ensure that genetic structure revealed by the PCA analyses of the complete dataset for individuals of Murrumbidgee ancestry (Fig 3 of the main manuscript) is not biased by physical linkage of loci, family structure, or isolation-by-distance effect, we conducted a set of analyses on the dataset of 73 individuals assigned to “adults” category. These were 70 individuals of length >271 mm at capture, and three individuals <250mm sampled in 2007- 2011, and thus not assigned to juvenile cohorts. We also used the dataset for all individuals of Murrumbidgee ancestry to test for asymmetric downstream dispersal by estimating immigration rates among sites.

First, we investigated the effect of physical linkage on our results. Mapping of DArT-tags to the published draft Macquarie perch genome (Pavlova et al. 2022) was performed by DArT using *blast*. We used PopLDdecay (Zhang et al. 2018) to calculate linkage disequilibrium (LD) decay using data for “adults” for three sites with samples sizes >10 (site 5, N=18; site 8, N=13; and site 9, N=17), following instructions in the manual for *SambaR* (de Jong et al. 2021). This plot (Figure C1) showed that LD (r^2^) drops to background levels at ~50 Kb, which is consistent with our studies of other organisms (Robledo-Ruiz et al. 2022).

Next, we used *SambaR* to filter out individuals missing >10% of data and loci missing >10% scores and to remove all but one locus that were within 50 Kb of each other (function *'filterdata'* of the R package *SambaR*, with indmiss=0.1, snpmiss=0.1, min_mac=1, dohefilter=FALSE and min_spacing=50000). As a result, 72 out of 73 individuals were retained, 1756 out of 1847 snps were retained after filtering, and 1607 after thinning (that is, only 8.5% of all loci retained after filtering were in moderate to high LD). This dataset was used to conduct principal coordinate analyses (PCoA) and Admixture analyses, and to calculate pairwise population *F*_ST_ values in *SambaR*. We then used *gl.drop.loc* function of *dartR* to remove loci filtered and thinned in the above *SambaR* analyses from the dataset for all individuals of Murrumbidgee and admixed ancestry. This dataset was used for the calculation of immigration rates, admixture (excluding individuals of admixed ancestry), and sibship.

**PCoAs** were performed using the function *pcoa* of the R package *ape-5.8* (Paradis and Schliep 2018) on Nei's genetic distances, calculated with the function *stamppNeisD* of the R package *StAMPP-1.6.3* (Pembleton, Cogan, and Forster 2013). Results for adults (Fig. C2) showed the same structure as the complete dataset (Fig. 3), where upstream sites 1-4 are isolated from downstream sites 5-12, some infrequent exchange of migrants occurs between site 5 and downstream sites, and sites 7-9 are in gene flow.

**Admixture analysis** was run through the *snmf* function of the R package *LEA* (Frichot et al. 2014). This function calculates least-squares estimates of ancestry proportions and ancestral allelic frequencies. It also calculates an entropy criterion that evaluates the quality of fit of the statistical model to the data by using a cross-validation technique, enabling to identify the number of ancestral genetic clusters (Frichot et al. 2014). In agreement with PCA analyses of all individuals (Fig. 3) or adults only (Fig. C2), the cross-entropy criterion for Macquarie perch data on Murrumbidgee adults was lowest at 3 (Fig. C3), suggesting 3 ancestral clusters, corresponding to sites 1-4, 5 and 6-12 (Fig. C4). K=4 also had a low value of the criterion, further separating sites 6-9 from sites 10-12, the latter thought to comprise non-breeding dispersers from the upstream sites. K=5 further separated site 1 from 2-4. Analysis of all individuals showed concordant results, with K=5 being the best number of clusters, except site 4 individuals formed an additional cluster (Figs. C5 and C6).

**Pairwise-population *F*_ST_-values** were calculated for adults with the functions *runWrightFst* of the R package *SambaR* (Table C1). Isolation-by-distance (IBD) analysis was performed with function *gl.ibd* of *dartR* package using geographic (river distances) and genetic (*F*_ST_) distance matrices as input (Table C1). Mantel test with 999 permutations showed significant effect of river distance on genetic differentiation (Mantel r=0.6232; P=0.001), explaining ~39% of the total variance in pairwise population *F*_ST_ values. Notwithstanding general pattern of IBD, genetic distances at short inter-site geographic distances were highly variable. We note that sample sizes were low for some populations. We also note that *F*_ST_ values do not account for asymmetric gene flow and differences in *N*e among sites, likely present in our system, and thus do not reflect true among-site differentiation.

**Immigration rates** between sites in the last two generations (i.e. ~14 years prior to 2023) were estimated in *BA3‐SNPs* (Mussmann et al. 2019) with default settings, using instructions provided in *SambaR* manual. Admixed individuals were removed. We ran the analysis twice: the first time all individuals of Murrumbidgee-only ancestry were grouped according to sampling sites 1-12, the second time apparently panmictic sites (Fig. 3) were pooled to have 7 groups: site 1, sites 2+3, site 4, site 5, site 6, sites 7+8+9 and sites 10 to 12.  Overall migration was low, and mainly restricted to neighbouring sites. Per-site analysis (Table C2) revealed some downstream migration from site 1 to 2, from 5 to 6, and from 8 to 9, and upstream migration from site 3 to 2, from 4 to 3, and from 8 to 7 and 6. Over 30% of individuals in sites 6, 7 and 9 appear to be derived from migrants from upstream and/or downstream sites. In contrast, less than 10% of individuals from sites 5 and 8 are migrants, but these sites appear be sources of migrants to other sites. The analysis of the seven approximately panmictic groups (Table C3) showed a consistent migration pattern; it also showed that 98% of individuals from pooled sites 7+8+9 are locally-born, and indicated dispersal from sites 2+3 to site 1 (in the upstream direction).

**Figure C1.** **Plot of linkage disequilibrium (LD) decay.** Linkage disequilibrium decay, as measured by pairwise r^2^ against physical distance between pair of loci. We identified 50 kb as the inflection point for linkage decay to be used as the window size to filter out physically closely-linked SNPs in *SambaR*.


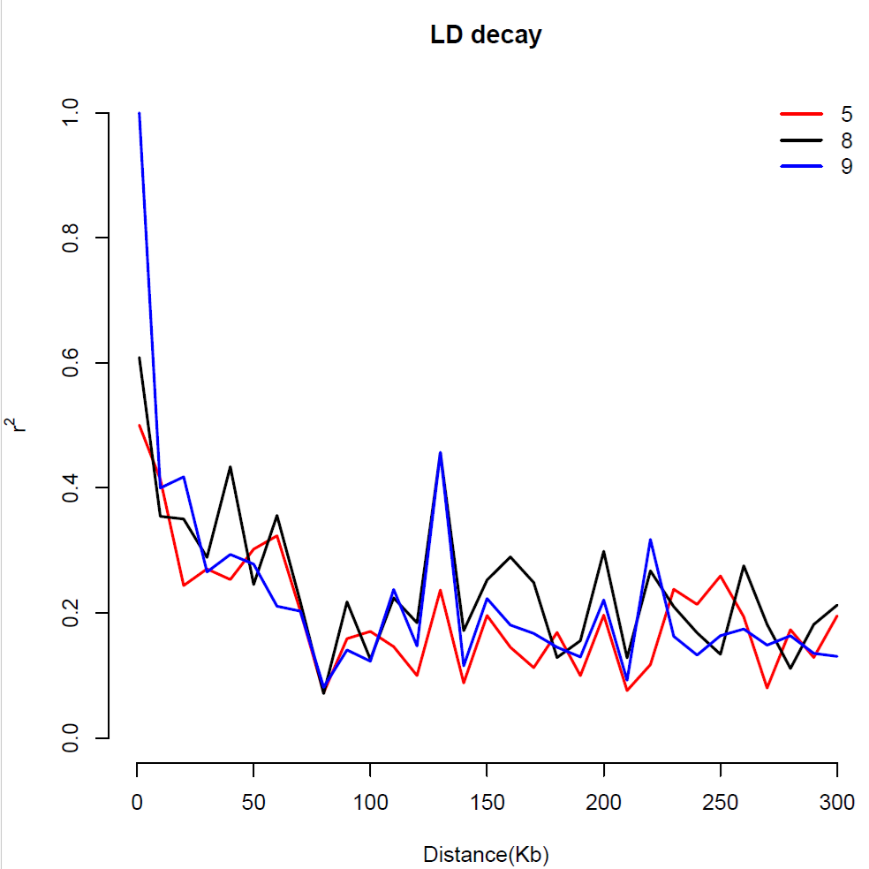


**Figure C2.** PCoA analysis of genotypes of adult. Colours as on Fig. 1 and Fig. 3 of the main manuscript.

**
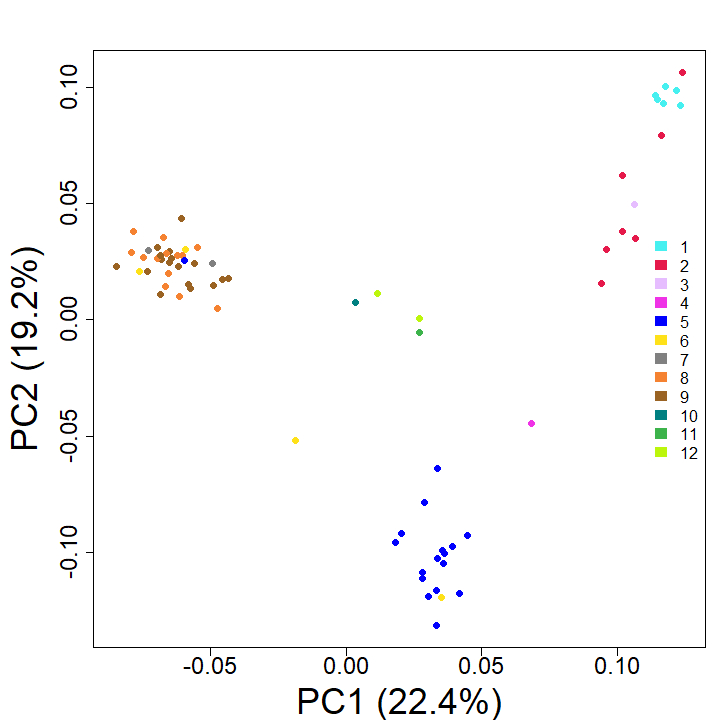
**

**Figure C3.** Cross-entropy criterion calculated by admixture analysis of adults.

**Figure C4.** Structure plot from analysis of genotypes of adults (assuming from 2 to 6 genetic clusters, K). Individuals (vertical lines) are arranged in order of sites, from upstream (site 1) to downstream (site 12).

**Figure C5.** Cross-entropy criterion calculated by admixture analysis of all individuals of Murrumbidgee-only ancestry in *LEA* (admixed individuals excluded).


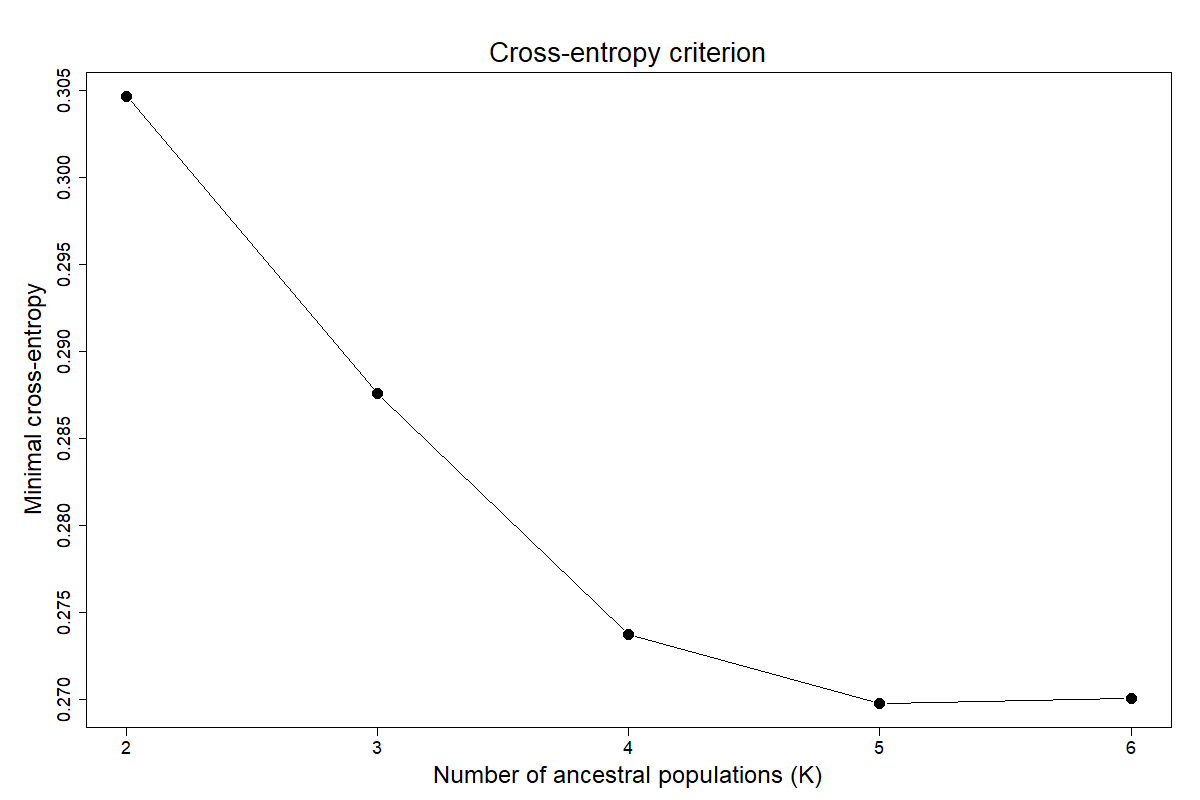


**Figure C6.** Structure plot from analysis of all individuals of Murrumbidgee-only ancestry (for K=5) cut into per-site samples and mapped over their respective sites.

**
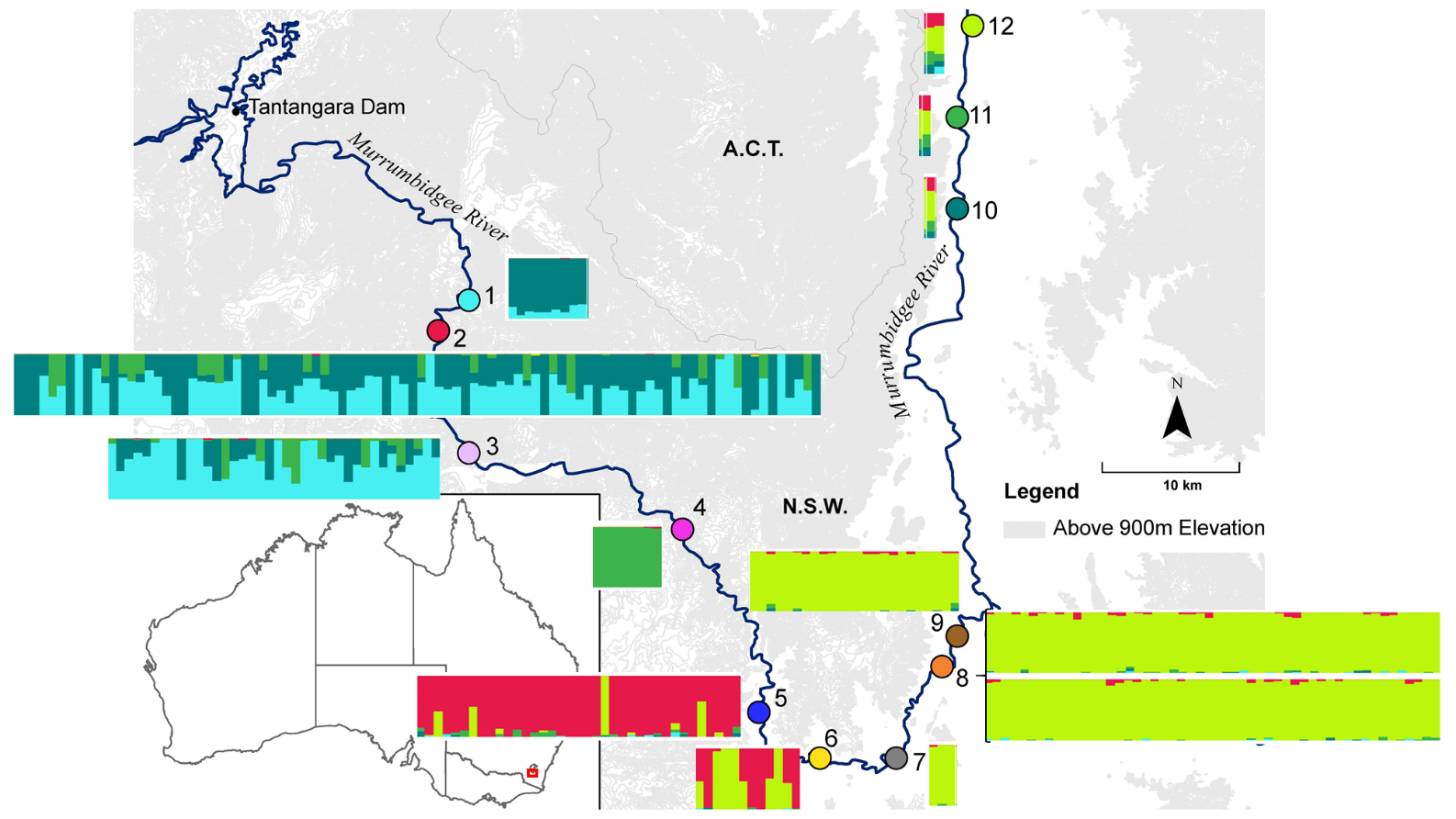
**

**Figure C7.** Isolation-by-distance plot showing significant relationship (P<0.05) between pairwise population *F*_ST_ values (Dgen) and river distances (Dgeo) for the adults (Table C1). **
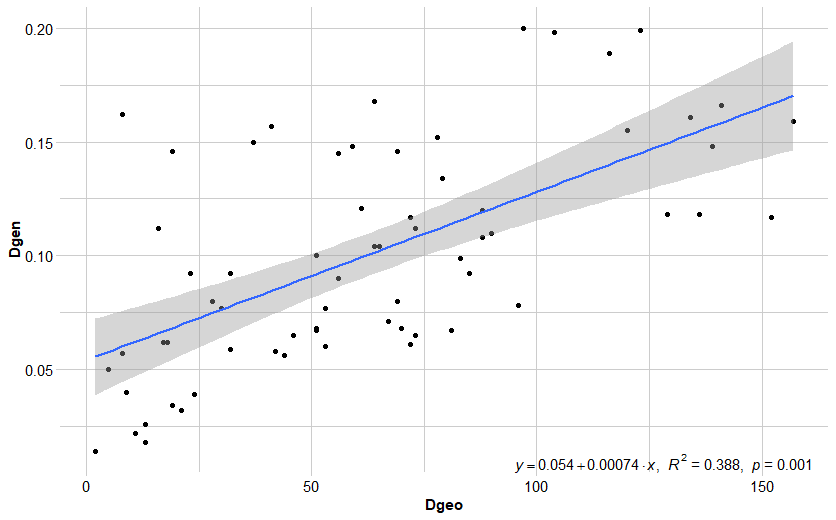
**

**Table C1.** Wright’s *F*_ST_-values (below diagonal) versus river distances, km (above diagonal) for 12 sampling sites (Fig. 1 of the main manuscript) calculated from adults. Sample sizes per site are given in the last column and the last row. Greener shading indicates smaller genetic or geographic distances, redder colours- larger distances.

| Site | site1 | site2 | site3 | site4 | site5 | site6 | site7 | site8 | site9 | site10 | site11 | site12 | Sample sizes |
| --- | --- | --- | --- | --- | --- | --- | --- | --- | --- | --- | --- | --- | --- |
| site1 | 0.0 | 5 | 18 | 37 | 61 | 69 | 78 | 88 | 90 | 134 | 141 | 157 | 6 |
| site2 | 0.05 | 0 | 13 | 32 | 56 | 64 | 73 | 83 | 85 | 129 | 136 | 152 | 7 |
| site3 | 0.06 | 0.03 | 0 | 19 | 42 | 51 | 59 | 70 | 72 | 116 | 123 | 139 | 1 |
| site4 | 0.15 | 0.06 | 0.15 | 0 | 24 | 32 | 41 | 51 | 53 | 97 | 104 | 120 | 1 |
| site5 | 0.12 | 0.09 | 0.06 | 0.04 | 0 | 9 | 17 | 28 | 30 | 73 | 81 | 96 | 18 |
| site6 | 0.15 | 0.10 | 0.10 | 0.09 | 0.04 | 0 | 8 | 19 | 21 | 65 | 72 | 88 | 4 |
| site7 | 0.15 | 0.11 | 0.15 | 0.16 | 0.06 | 0.06 | 0 | 11 | 13 | 56 | 64 | 79 | 2 |
| site8 | 0.12 | 0.10 | 0.07 | 0.07 | 0.08 | 0.03 | 0.02 | 0 | 2 | 46 | 53 | 69 | 13 |
| site9 | 0.11 | 0.09 | 0.06 | 0.06 | 0.08 | 0.03 | 0.02 | 0.01 | 0 | 44 | 51 | 67 | 17 |
| site10 | 0.16 | 0.12 | 0.19 | 0.20 | 0.07 | 0.10 | 0.15 | 0.07 | 0.06 | 0 | 8 | 23 | 1 |
| site11 | 0.17 | 0.12 | 0.20 | 0.20 | 0.07 | 0.12 | 0.17 | 0.08 | 0.07 | 0.16 | 0 | 16 | 1 |
| site12 | 0.16 | 0.12 | 0.15 | 0.16 | 0.08 | 0.11 | 0.13 | 0.08 | 0.07 | 0.09 | 0.11 | 0 | 2 |
| Sample size | 6 | 7 | 1 | 1 | 18 | 4 | 2 | 13 | 17 | 1 | 1 | 2 | 73 |

**Table C2.** Results of BayesAss (BA3-SNPs) analysis of immigration rate using dataset for all Murrumbidgee individuals (except admixed ones) organized by sampling sites 1-12. The off-diagonal numbers are estimates of the fraction of individuals in the receiving population that are migrants derived from the source population (per generation). Diagonals (grey shading) indicate the proportion of individuals at a site that are not first- or second-generation migrants: the smaller these values, the larger proportion of migrants the population is receiving. Migration in the upstream direction is above the diagonal, in the downstream direction below the diagonal; immigration rates >0.05 are shaded yellow.

|  |  | From | From | From | From | From | From | From | From | From | From | From | From |
| --- | --- | --- | --- | --- | --- | --- | --- | --- | --- | --- | --- | --- | --- |
|  |  | site1 | site2 | site3 | site4 | site5 | site6 | site7 | site8 | site9 | site10 | site11 | site12 |
| To | site1 | 0.81 | 0.02 | 0.03 | 0.01 | 0.02 | 0.02 | 0.02 | 0.02 | 0.02 | 0.02 | 0.02 | 0.02 |
| To | site2 | 0.09 | 0.73 | 0.12 | 0.03 | 0.00 | 0.00 | 0.00 | 0.00 | 0.00 | 0.00 | 0.00 | 0.00 |
| To | site3 | 0.02 | 0.01 | 0.81 | 0.11 | 0.01 | 0.01 | 0.01 | 0.01 | 0.01 | 0.01 | 0.01 | 0.01 |
| To | site4 | 0.02 | 0.02 | 0.02 | 0.82 | 0.02 | 0.02 | 0.02 | 0.02 | 0.02 | 0.02 | 0.02 | 0.02 |
| To | site5 | 0.01 | 0.01 | 0.01 | 0.01 | 0.90 | 0.01 | 0.01 | 0.03 | 0.01 | 0.01 | 0.01 | 0.01 |
| To | site6 | 0.01 | 0.01 | 0.01 | 0.01 | 0.08 | 0.68 | 0.01 | 0.11 | 0.01 | 0.01 | 0.01 | 0.01 |
| To | site7 | 0.02 | 0.02 | 0.02 | 0.02 | 0.02 | 0.02 | 0.69 | 0.09 | 0.02 | 0.02 | 0.02 | 0.02 |
| To | site8 | 0.00 | 0.00 | 0.00 | 0.00 | 0.00 | 0.00 | 0.00 | 0.96 | 0.00 | 0.00 | 0.00 | 0.00 |
| To | site9 | 0.01 | 0.01 | 0.01 | 0.01 | 0.01 | 0.01 | 0.01 | 0.22 | 0.68 | 0.01 | 0.01 | 0.01 |
| To | site10 | 0.03 | 0.02 | 0.03 | 0.02 | 0.02 | 0.03 | 0.03 | 0.03 | 0.02 | 0.72 | 0.03 | 0.03 |
| To | site11 | 0.03 | 0.02 | 0.03 | 0.03 | 0.03 | 0.03 | 0.02 | 0.02 | 0.03 | 0.03 | 0.72 | 0.03 |
| To | site12 | 0.02 | 0.03 | 0.02 | 0.02 | 0.02 | 0.02 | 0.02 | 0.02 | 0.02 | 0.02 | 0.02 | 0.74 |

**Table C3.** Results of BayesAss (BA3-SNPs) analysis of immigration rates using a dataset organized by genetic clusters into site 1, sites 2-3, site 4, site 5, site 6, sites 7-9, sites 10-12. The off-diagonal numbers are estimates of the fraction of individuals in the receiving population that are migrants derived from the source population (per generation). Diagonals (grey shading) indicate the proportion of individuals at a site that are not first- or second-generation migrants: the smaller these values the larger proportion of migrants the population is receiving. Migration in upstream direction is above diagonal, in the downstream direction below the diagonal; immigration rates >0.05 are shaded yellow.

|  |  | From | From | From | From | From | From | From |
| --- | --- | --- | --- | --- | --- | --- | --- | --- |
|  |  | site1 | site2.3 | site4 | site5 | site6 | sites7.8.9 | sites10.11.12 |
| To | site1 | 0.81 | 0.09 | 0.02 | 0.02 | 0.02 | 0.02 | 0.02 |
| To | site2.3 | 0.11 | 0.79 | 0.09 | 0.00 | 0.00 | 0.00 | 0.00 |
| To | site4 | 0.02 | 0.02 | 0.87 | 0.02 | 0.02 | 0.02 | 0.02 |
| To | site5 | 0.01 | 0.01 | 0.01 | 0.92 | 0.01 | 0.04 | 0.01 |
| To | site6 | 0.02 | 0.02 | 0.02 | 0.09 | 0.68 | 0.15 | 0.02 |
| To | sites7.8.9 | 0.00 | 0.00 | 0.00 | 0.00 | 0.00 | 0.98 | 0.00 |
| To | sites10.11.12 | 0.03 | 0.03 | 0.03 | 0.03 | 0.03 | 0.03 | 0.82 |

# **Appendix D.** Identity and sibship analyses in *Colony2*.

Preliminary identity analysis was run using the dataset not filtered for physical linkage, for all individuals sampled in the Murrumbidgee except MP_M206 of Cataract Reservoir origin, scored for 3,098 SNPs. This analysis showed two pairs of genotypes to be identical: MP_M111 and MP_M160, and M279 and MP_M248. Final identity and sibship analyses were run on a filtered (including for minor allele count <2) and thinned (resulting in loci not in the same 50 Kb windows) dataset split into the upstream (sites 1-4; 145 individuals scored for 964 SNPs) and downstream (sites 5-12; 180 individuals scored for 1,242 SNPs) sub-datasets. This was done to focus the analyses given the apparent isolation of these river fragments. One sample from each pair of identical genotypes were removed (MP_M160 and MP_M248) from final sibship analysis.

Results of **identity analysis** showed that all seven pairs of identical genotypes were sampled at the same site from one to seven years apart, consistent with individuals being sampled twice (Table D1). **Sibship** analysis in *Colony2* showed that 169 of 320 unique individuals (52.8%) were arranged in 28 families of two or more individuals, with the largest four families (family IDs 1-4) containing 16-30 full-siblings (Table D2). Full-sibs from 18 families (including the three largest ones) were sampled from the same site, whilst those from 10 families were sampled from two sites: two––families 5 and 13––from sites 1-2, four––families 4, 9, 12, 14–– from sites 2-3, one––family 7––from sites 5-6, two––families 23 and 24––from sites 8-9 and one––family 28––from sites 10-12. Twelve families comprised individuals born in different cohorts; of these three (families 2, 4, 5) were born in three consecutive years, three (7, 9, 12) in two non-consecutive years and six (families 1, 8, 10, 14, 17, 24) in consecutive years (here, individuals captured as adults are ignored).

Sibship analysis revealed that two of three admixed individuals were full-siblings (MP_M287 born 2021, captured in 2023 at site 9 and MP_M320 born 2022, captured at site 8 in 2023). The third admixed individual MP_M325 born 2021, captured at site 8 in 2023, was identified as their half-sib in all five replicates (with probability=1). Therefore, one or two translocated Cataract Reservoir fish successfully bred in the Murrumbidgee, including one that bred with the same local partner for two consecutive years.

**Table D1**. Identity analysis: Sample1 and Sample2 are identifiers of identical genotypes.

| **Sample1** | **Sample1 site and birth cohort** | **Sample1 sampling year/age** | **Sample2** | **Sample2 site and birth cohort** | **Sample2 sampling date/age** |
| --- | --- | --- | --- | --- | --- |
| MP_M111 | Site2.2019 | 2020/0-1YO | MP_M160 | Site2.2019 | 2021/1-2YO |
| MP_M248 | Site2.2019 | 2022/2-3YO | MP_M279 | Site2.NA | 2023/adult |
| MP_M250 | Site2.2020 | 2022/1-2YO | MP_M280 | Site2.2020 | 2023/2-3YO |
| MP_M246 | Site2.2019 | 2022/2-3YO | MP_M282 | Site2.NA | 2023/adult |
| MP_M245 | Site2.2020 | 2022/1-2YO | MP_M281 | Site2.2020 | 2023/2-3YO |
| MP_M93 | Site8.2019 | 2020/0-1YO | MP_M228 | Site8.2019 | 2021/1-2YO |
| MP_M82 | Site9.NA | 2014/adult | AR7237282 | Site9.NA | 2021/adult |

**Table D2.** Distribution of inferred full-sib members of Murrumbidgee Macquarie perch across birth-year cohorts and sites for 28 families of two or more members. Full-sib family IDs are sorted by number of full-sibs per family. Identical genotypes are excluded from the total count.

| **Family ID** | **Site** | **2018** | **2019** | **2020** | **2021** | **2022** | **Adults** | **Number of full-sibs per family** |
| --- | --- | --- | --- | --- | --- | --- | --- | --- |
| 1 | 5 |  | 5 | 10 |  |  | 15 | 30 |
| 2 | 2 | 6 | 9 | 4 |  |  | 1 | 20 |
| 3 | 2 |  |  | 18 |  |  |  | 18 |
| 4 | 2 | 2 | 1 | 2 |  |  |  | 5 |
|  | 3 |  | 1 | 10 |  |  |  | 11 |
| 5 | 1 |  |  | 3 |  |  | 3 | 6 |
|  | 2 |  | 1 |  | 1 |  |  | 2 |
| 6 | 4 |  |  | 7 |  |  | 1 | 8 |
| 7 | 5 |  | 1 |  |  |  | 2 | 3 |
|  | 6 |  |  |  |  | 3 | 1 | 4 |
| 8 | 2 | 3 | 3 |  |  |  |  | 6 |
| 9 | 2 | 1 |  | 1 |  |  |  | 2 |
|  | 3 |  |  | 4 |  |  |  | 4 |
| 10 | 2 | 4 | 1 |  |  |  | 1 | 6 |
| 11 | 2 |  |  | 6 |  |  |  | 6 |
| 12 | 2 | 1 |  |  |  |  |  | 1 |
|  | 3 |  |  | 5 |  |  |  | 5 |
| 13 | 1 |  |  |  |  |  | 3 | 3 |
|  | 2 |  | 1 |  |  |  |  | 1 |
| 14 | 2 | 1 |  |  |  |  |  | 1 |
|  | 3 |  | 1 |  |  |  |  | 1 |
| 15 | 2 |  |  |  |  |  | 2 | 2 |
| 16 | 2 |  | 2 |  |  |  |  | 2 |
| 17 | 2 | 1 | 1 |  |  |  |  | 2 |
| 18 | 3 |  |  | 2 |  |  |  | 2 |
| 19 | 8 |  |  | 2 |  |  |  | 2 |
| 20 | 8 |  |  | 2 |  |  |  | 2 |
| 21 | 8 |  |  | 2 |  |  |  | 2 |
| 22 | 6 |  |  |  |  | 2 |  | 2 |
| 23 | 8 |  |  |  |  | 1 |  | 1 |
|  | 9 |  |  |  |  | 1 |  | 1 |
| 24 | 8 |  |  |  |  | 1 |  | 1 |
|  | 9 |  |  |  | 1 |  |  | 1 |
| 25 | 8 |  |  |  |  | 2 |  | 2 |
| 26 | 8 |  |  | 2 |  |  |  | 2 |
| 27 | 5 |  |  | 2 |  |  |  | 2 |
| 28 | 10 |  |  |  |  |  | 1 | 1 |
|  | 12 |  |  |  |  |  | 1 | 1 |

**Figure D1.** Distribution of the 12 largest families (N>4) of Murrumbidgee Macquarie perch in genotypic PCA space (see Fig. 3 of the main manuscript for the same points coloured by site). Colours represent families 1-12. See Fig. 3 of the main manuscript, where the same points (individuals) are coloured according to the sampling site. NAs (grey points) represent individuals for which Colony2 failed to consistently determine full-sibs across 5 replicates.


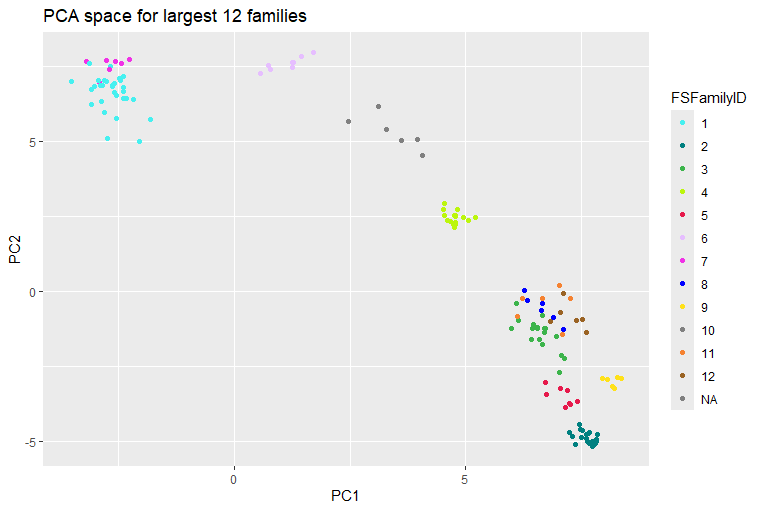


# **Appendix E.** Estimates of genetic diversity and effective number of breeders for various groups

**Table E1.** Mean heterozygosity (PHt) and standard deviations for different ancestries (Fig. E1), sites (Fig. E2), cohorts (Fig. E4) and site-per-cohort (Fig. E5; significant Tukey tests in Table E2).

| **Ancestry** | **mean PHt** | **sd** | **N** |  | **Site-per-cohort** | **mean PHt** | **sd** | **N** |
| --- | --- | --- | --- | --- | --- | --- | --- | --- |
| Admixed | 0.207 | 0.006 | 3 |  | Site 1.2020 | 0.077 | 0.007 | 3 |
| Cataract Reservoir | 0.165 | 0.024 | 68 |  | Site 1.adult | 0.077 | 0.006 | 6 |
| Murrumbidgee River | 0.098 | 0.022 | 326 |  | Site 2.2018 | 0.095 | 0.025 | 24 |
| **Site** | **mean PHt** | **sd** |  |  | Site 2.2019 | 0.082 | 0.022 | 25 |
| Site 1 | 0.077 | 0.006 | 9 |  | Site 2.2020 | 0.104 | 0.027 | 35 |
| Site 2 | 0.095 | 0.026 | 92 |  | Site 2.2021 | 0.078 | NA | 1 |
| Site 3 | 0.109 | 0.032 | 38 |  | Site 2.adult | 0.096 | 0.018 | 7 |
| Site 4 | 0.078 | 0.005 | 8 |  | Site 3.2018 | 0.085 | NA | 1 |
| Site 5 | 0.107 | 0.019 | 37 |  | Site 3.2019 | 0.121 | 0.040 | 2 |
| Site 6 | 0.119 | 0.027 | 12 |  | Site 3.2020 | 0.108 | 0.033 | 33 |
| Site 7 | 0.120 | 0.015 | 3 |  | Site 3.2021 | 0.117 | NA | 1 |
| Site 8 | 0.121 | 0.019 | 100 |  | Site 3.adult | 0.126 | NA | 1 |
| Site 9 | 0.120 | 0.025 | 25 |  | Site 4.2020 | 0.077 | 0.005 | 7 |
| Site 10 | 0.121 | NA | 1 |  | Site 4.adult | 0.083 | NA | 1 |
| Site 11 | 0.109 | NA | 1 |  | Site 5.2019 | 0.101 | 0.019 | 7 |
| Site 12 | 0.126 | 0.006 | 2 |  | Site 5.2020 | 0.118 | 0.014 | 12 |
| **Cohort** | **mean PHt** | **sd** |  |  | Site 5.adult | 0.102 | 0.020 | 18 |
| 2017 | 0.120 | NA | 1 |  | Site 6.2020 | 0.131 | 0.014 | 3 |
| 2018 | 0.098 | 0.024 | 31 |  | Site 6.2022 | 0.109 | 0.035 | 5 |
| 2019 | 0.093 | 0.025 | 40 |  | Site 6.adult | 0.122 | 0.026 | 4 |
| 2020 | 0.110 | 0.024 | 144 |  | Site 7.2022 | 0.118 | NA | 1 |
| 2021 | 0.138 | 0.057 | 8 |  | Site 7.adult | 0.120 | 0.021 | 2 |
| 2022 | 0.121 | 0.024 | 30 |  | Site 8.2017 | 0.120 | NA | 1 |
| adult | 0.109 | 0.022 | 74 |  | Site 8.2018 | 0.115 | 0.005 | 6 |
|  |  |  |  |  | Site 8.2019 | 0.120 | 0.005 | 6 |
|  |  |  |  |  | Site 8.2020 | 0.117 | 0.008 | 47 |
|  |  |  |  |  | Site 8.2021 | 0.149 | 0.073 | 3 |
|  |  |  |  |  | Site 8.2022 | 0.125 | 0.022 | 23 |
|  |  |  |  |  | Site 8.adult | 0.125 | 0.024 | 14 |
|  |  |  |  |  | Site 9.2020 | 0.114 | 0.008 | 4 |
|  |  |  |  |  | Site 9.2021 | 0.156 | 0.060 | 3 |
|  |  |  |  |  | Site 9.2022 | 0.106 | NA | 1 |
|  |  |  |  |  | Site 9.adult | 0.115 | 0.015 | 17 |
|  |  |  |  |  | Site 10.adult | 0.121 | NA | 1 |
|  |  |  |  |  | Site 11.adult | 0.109 | NA | 1 |
|  |  |  |  |  | Site 12.adult | 0.126 | 0.006 | 2 |

**Table E2.** Results of significant (P<0.05) posthoc Tukey’s HSD test for comparisons of Murrumbidgee Macquarie perch heterozygosity between (a) sites (model Heterozygosity ~ Site; Fig. E2); (b) cohorts (model Heterozygosity ~ Cohort; Fig. E4) or (c) site per cohorts (model Heterozygosity ~Site-by-Cohort; only categories with N>10 were tested; Fig. E5 includes all categories) for the Murrumbidgee data. Diff- difference between the means of the two groups being compared (value >0 indicates that the first group has a higher mean than the second group); lwr- the lower bound of the confidence interval for the difference between the group; upr- the upper bound of the confidence interval for the difference between the group means; p adj- P-value adjusted for multiple comparisons using the Tukey method.

| **Pair of sites** | **diff** | **lwr** | **upr** | **p adj** |
| --- | --- | --- | --- | --- |
| Sites 3-1 | 0.032 | 0.004 | 0.06 | 0.013 |
| Sites 5-1 | 0.03 | 0.001 | 0.058 | 0.032 |
| Sites 6-1 | 0.041 | 0.008 | 0.075 | 0.004 |
| Sites 8-1 | 0.044 | 0.017 | 0.07 | 0 |
| Sites 9-1 | 0.042 | 0.013 | 0.072 | 0 |
| Sites 6-2 | 0.024 | 0 | 0.047 | 0.043 |
| Sites 8-2 | 0.026 | 0.015 | 0.037 | 0 |
| Sites 9-2 | 0.025 | 0.008 | 0.042 | 0 |
| Sites 4-3 | -0.032 | -0.061 | -0.002 | 0.026 |
| Sites 6-4 | 0.041 | 0.006 | 0.076 | 0.007 |
| Sites 8-4 | 0.044 | 0.015 | 0.072 | 0 |
| Sites 9-4 | 0.042 | 0.011 | 0.073 | 0.001 |
| **Pair of cohorts** | **diff** | **lwr** | **upr** | **p adj** |
| 2021-2018 | 0.040 | 0.011 | 0.069 | 0.001 |
| 2022-2018 | 0.023 | 0.004 | 0.042 | 0.007 |
| 2020-2019 | 0.016 | 0.003 | 0.030 | 0.005 |
| 2021-2019 | 0.045 | 0.017 | 0.074 | 0.000 |
| 2022-2019 | 0.028 | 0.010 | 0.046 | 0.000 |
| adult-2019 | 0.016 | 0.002 | 0.031 | 0.016 |
| 2021-2020 | 0.029 | 0.002 | 0.056 | 0.025 |
| adult-2021 | -0.029 | -0.057 | -0.002 | 0.031 |
| **Pair of site-per-cohorts** | **diff** | **lwr** | **upr** | **p adj** |
| Site 8.2020- Site 2.2018 | 0.023 | 0.006 | 0.039 | 0.001 |
| Site 8.2022- Site 2.2018 | 0.026 | 0.006 | 0.046 | 0.002 |
| Site 8.adult- Site 2.2018 | 0.024 | 0.001 | 0.047 | 0.029 |
| Site 2.2020- Site 2.2019 | 0.022 | 0.004 | 0.039 | 0.004 |
| Site 3.2020- Site 2.2019 | 0.026 | 0.008 | 0.044 | 0.000 |
| Site 5.2020- Site 2.2019 | 0.036 | 0.012 | 0.059 | 0.000 |
| Site 8.2020- Site 2.2019 | 0.035 | 0.018 | 0.052 | 0.000 |
| Site 8.2022- Site 2.2019 | 0.038 | 0.019 | 0.058 | 0.000 |
| Site 8.adult- Site 2.2019 | 0.037 | 0.014 | 0.060 | 0.000 |
| Site 9.adult- Site 2.2019 | 0.033 | 0.012 | 0.054 | 0.000 |

**Table E3.** Population heterozygosity, calculated for all 45 Cataract Reservoir fish and all 328 fish captured in the Murrumbidgee River (regardless of their ancestry). N inds with data- sample size accounting for missing genotypes; N loci- number of loci analyzed; N poly loci- number of polymorphic loci; Ho- observed heterozygosity averaged across loci; HoSD- standard deviation of observed heterozygosity across loci; He- expected heterozygosity, averaged across loci; HeSD- standard deviation of expected heterozygosity across loci; FIS- **inbreeding coefficient,** measuring the reduction in observed heterozygosity within a population compared to what is expected under Hardy-Weinberg equilibrium (values >0 suggest a deficit of heterozygotes, which might indicate inbreeding and/or population structure).

| **Population** | **N inds with data** | **N loci** | **N poly loci** | **Ho** | **HoSD** | **He** | **HeSD** | ***F*_IS_** |
| --- | --- | --- | --- | --- | --- | --- | --- | --- |
| Cataract Reservoir | 44.9 | 3447 | 2074 | 0.163 | 0.175 | 0.181 | 0.186 | 0.108 |
| Murrumbidgee River | 326.4 | 3447 | 3182 | 0.100 | 0.142 | 0.128 | 0.179 | 0.219 |

**Figure E1.** Individual heterozygosity (PHt) from 3447 SNPs scored from all 375 individuals, averaged per ancestry. All other estimates of heterozygosity (below) were conducted on the dataset of 3173 SNPs scored for 328 individuals (of all ancestries) captured in the Murrumbidgee.


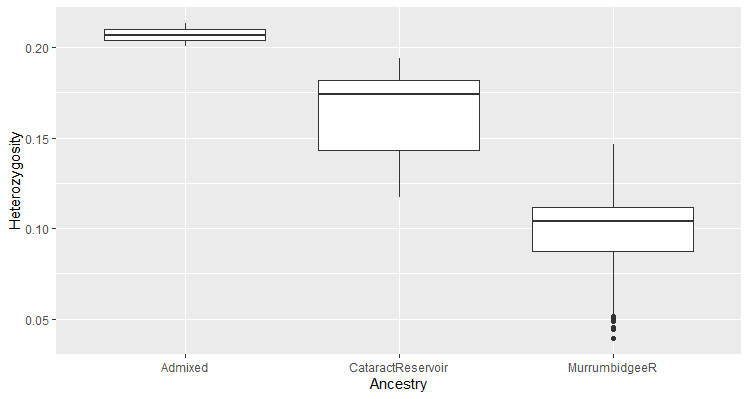


**Figure E2.** Scatterplot showing the relationship between downstream river distance from the most upstream site 1 and individual heterozygosity (PHt, from 3173 SNPs scored for 328 individuals of all ancestries captured in the Murrumbidgee). This relationship is significant: adjusted R-squared = 0.1772, P<0.001.

**
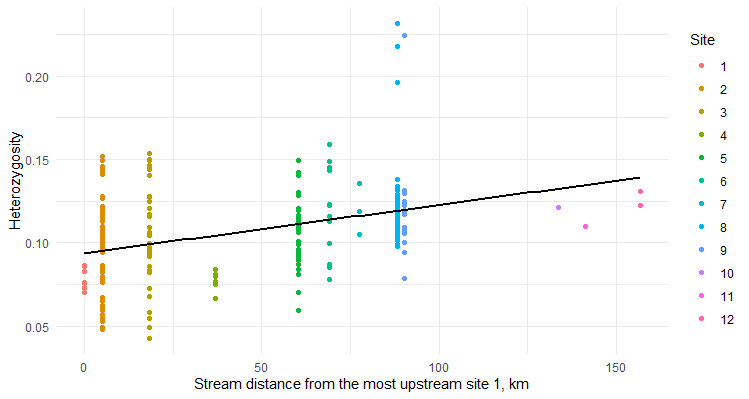
**

**Figure E3.** Individual heterozygosity (PHt; from 3173 SNPs scored for 328 individuals of all ancestries captured in the Murrumbidgee), averaged per site (cohorts pooled). The three individuals with PHt>0.2 are admixed, the individual with PHt=0.195 is a recaptured Cataract Reservoir individual.


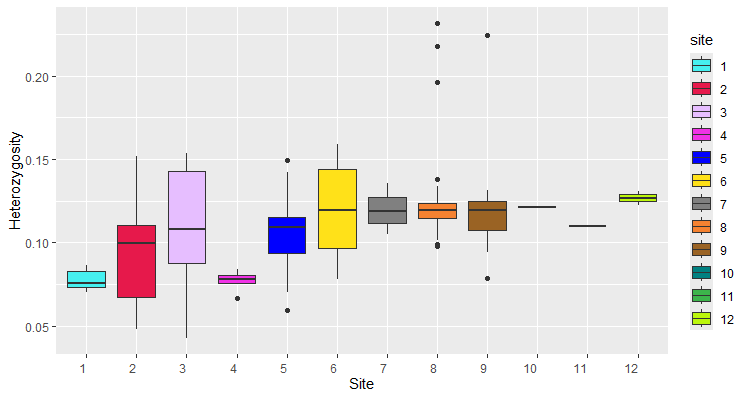


**Figure E4** Individual heterozygosity, averaged per cohort (sites pooled). The three individuals with PHt>0.2 are admixed, the individual with PHt=0.195 is a recaptured Cataract Reservoir individual (assigned to adults).


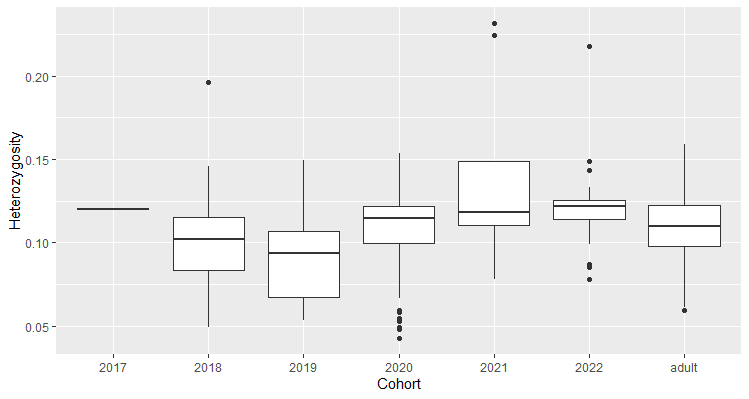


**Figure E5.** Individual heterozygosity of Murrumbidgee Macquarie perch averaged per site per cohort. Sample sizes are above bars.


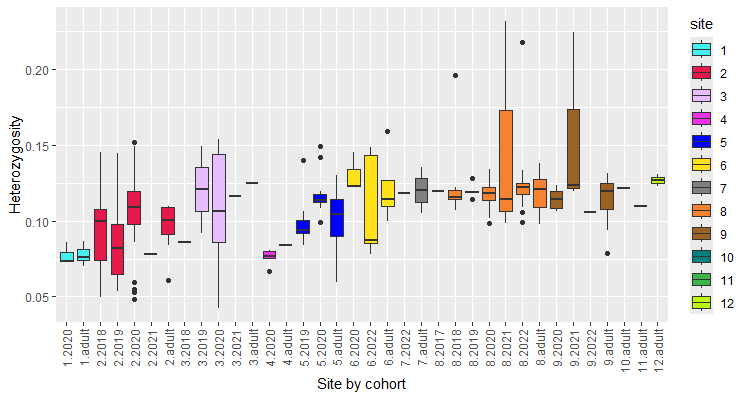

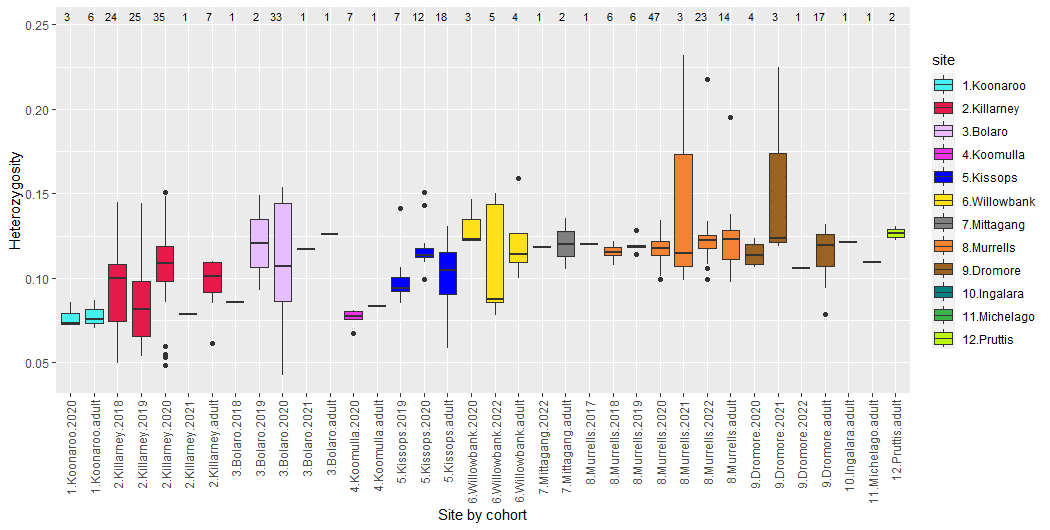


**Appendix F.** Results of linear models (LM) modelling Murrumbidgee Macquarie perch heterozygosity as a function of site and cohort for juveniles born 2018-onwards. Plot is on Fig. 4 of the main text.

**Table F1.** Results of linear models testing the effect of population structure (approximated by site, LM.H.site), environmental variation during birth year (approximated by cohort, LM.H.cohort) or both (LM.H.site.cohort) explain variation in heterozygosity (PHt). LM.H.site: PHt ~ site; LM.H.cohort: PHt ~ cohort; LM.H.site.cohort: PHt ~ cohort + site; LM.H.site*cohort: PHt~ cohort*site. P<0.05 are in red. Flow cannot be used as a variable due to being confounded with cohort.

| **Model** | **Term** | **Estimate** | **Std.Error** | **t value** | **Pr(>\|t\|)** | **Adjusted R-squared** | **P-value** |
| --- | --- | --- | --- | --- | --- | --- | --- |
| LM.H.site | (Intercept) | 0.077 | 0.012 | 6.176 | 0.000 | 0.205 | 0.000 |
| PHt ~ site | Site2 | 0.018 | 0.013 | 1.396 | 0.164 |  |  |
|  | Site3 | 0.032 | 0.013 | 2.440 | 0.015 |  |  |
|  | Site4 | 0.000 | 0.015 | -0.023 | 0.982 |  |  |
|  | Site5 | 0.035 | 0.013 | 2.582 | 0.010 |  |  |
|  | Site6 | 0.040 | 0.015 | 2.735 | 0.007 |  |  |
|  | Site7 | 0.041 | 0.025 | 1.658 | 0.099 |  |  |
|  | Site8 | 0.041 | 0.013 | 3.225 | 0.001 |  |  |
|  | Site9 | 0.038 | 0.015 | 2.549 | 0.011 |  |  |
| LM.H.cohort | (Intercept) | 0.098 | 0.004 | 23.629 | 0.000 | 0.083 | 0.000 |
| PHt ~ cohort | Cohort2019 | -0.005 | 0.006 | -0.969 | 0.333 |  |  |
|  | Cohort2020 | 0.011 | 0.005 | 2.406 | 0.017 |  |  |
|  | Cohort2021 | 0.010 | 0.010 | 0.977 | 0.330 |  |  |
|  | Cohort2022 | 0.019 | 0.006 | 3.248 | 0.001 |  |  |
| LM.H.site.cohort | (Intercept) | 0.071 | 0.013 | 5.404 | 0.000 | 0.229 | 0.000 |
| PHt~site+cohort | Site2 | 0.024 | 0.013 | 1.860 | 0.064 |  |  |
|  | Site3 | 0.033 | 0.013 | 2.558 | 0.011 |  |  |
|  | Site4 | 0.000 | 0.015 | -0.023 | 0.981 |  |  |
|  | Site5 | 0.040 | 0.013 | 2.983 | 0.003 |  |  |
|  | Site6 | 0.041 | 0.015 | 2.782 | 0.006 |  |  |
|  | Site7 | 0.043 | 0.025 | 1.713 | 0.088 |  |  |
|  | Site8 | 0.043 | 0.013 | 3.409 | 0.001 |  |  |
|  | Site9 | 0.040 | 0.015 | 2.660 | 0.008 |  |  |
|  | Cohort2019 | -0.008 | 0.005 | -1.452 | 0.148 |  |  |
|  | Cohort2020 | 0.006 | 0.005 | 1.345 | 0.180 |  |  |
|  | Cohort2021 | 0.001 | 0.010 | 0.076 | 0.940 |  |  |
|  | Cohort2022 | 0.005 | 0.006 | 0.715 | 0.475 |  |  |
| LM.H.site*cohort | (Intercept) | 0.063 | 0.028 | 2.228 | 0.027 | 0.238 | 0.000 |
| PHt~site*cohort | Site2 | 0.032 | 0.029 | 1.103 | 0.271 |  |  |
|  | Site3 | 0.022 | 0.035 | 0.632 | 0.528 |  |  |
|  | Site4 | 0.000 | 0.015 | -0.024 | 0.981 |  |  |
|  | Site5 | 0.041 | 0.014 | 3.008 | 0.003 |  |  |
|  | Site6 | 0.041 | 0.028 | 1.436 | 0.152 |  |  |
|  | Site7 | 0.050 | 0.034 | 1.472 | 0.142 |  |  |
|  | Site8 | 0.052 | 0.027 | 1.934 | 0.054 |  |  |
|  | Site9 | 0.037 | 0.016 | 2.307 | 0.022 |  |  |
|  | Cohort2019 | 0.004 | 0.012 | 0.347 | 0.729 |  |  |
|  | Cohort2020 | 0.014 | 0.026 | 0.543 | 0.588 |  |  |
|  | Cohort2021 | 0.021 | 0.028 | 0.751 | 0.454 |  |  |
|  | Cohort2022 | 0.005 | 0.010 | 0.530 | 0.597 |  |  |
|  | Site2:Cohort2019 | -0.017 | 0.014 | -1.220 | 0.224 |  |  |
|  | Site3:Cohort2019 | 0.031 | 0.029 | 1.078 | 0.282 |  |  |
|  | Site4:Cohort2019 | NA | NA | NA | NA |  |  |
|  | Site5:Cohort2019 | -0.008 | 0.027 | -0.284 | 0.777 |  |  |
|  | Site6:Cohort2019 | NA | NA | NA | NA |  |  |
|  | Site7:Cohort2019 | NA | NA | NA | NA |  |  |
|  | Site8:Cohort2019 | NA | NA | NA | NA |  |  |
|  | Site9:Cohort2019 | NA | NA | NA | NA |  |  |
|  | Site2:Cohort2020 | -0.005 | 0.026 | -0.177 | 0.860 |  |  |
|  | Site3:Cohort2020 | 0.009 | 0.033 | 0.270 | 0.787 |  |  |
|  | Site4:Cohort2020 | NA | NA | NA | NA |  |  |
|  | Site5:Cohort2020 | NA | NA | NA | NA |  |  |
|  | Site6:Cohort2020 | 0.013 | 0.028 | 0.461 | 0.645 |  |  |
|  | Site7:Cohort2020 | NA | NA | NA | NA |  |  |
|  | Site8:Cohort2020 | -0.012 | 0.024 | -0.488 | 0.626 |  |  |
|  | Site9:Cohort2020 | NA | NA | NA | NA |  |  |
|  | Site2:Cohort2021 | -0.037 | 0.035 | -1.061 | 0.290 |  |  |
|  | Site3:Cohort2021 | 0.011 | 0.041 | 0.267 | 0.789 |  |  |
|  | Site4:Cohort2021 | NA | NA | NA | NA |  |  |
|  | Site5:Cohort2021 | NA | NA | NA | NA |  |  |
|  | Site6:Cohort2021 | NA | NA | NA | NA |  |  |
|  | Site7:Cohort2021 | NA | NA | NA | NA |  |  |
|  | Site8:Cohort2021 | -0.029 | 0.030 | -0.971 | 0.333 |  |  |
|  | Site9:Cohort2021 | NA | NA | NA | NA |  |  |
|  | Site2:Cohort2022 | NA | NA | NA | NA |  |  |
|  | Site3:Cohort2022 | NA | NA | NA | NA |  |  |
|  | Site4:Cohort2022 | NA | NA | NA | NA |  |  |
|  | Site5:Cohort2022 | NA | NA | NA | NA |  |  |
|  | Site6:Cohort2022 | NA | NA | NA | NA |  |  |
|  | Site7:Cohort2022 | NA | NA | NA | NA |  |  |
|  | Site8:Cohort2022 | NA | NA | NA | NA |  |  |
|  | Site9:Cohort2022 | NA | NA | NA | NA |  |  |

# **Appendix G.** Results of linear models (LM) of Murrumbidgee Macquarie perch growth residuals as a function of site, cohort and heterozygosity for juveniles born 2018-onwards (Table G1), posthoc Tukey tests for models LM.G.site and LM.G.cohort (Tables G2-G3) and plots of growth residuals by sites (Fig. G1), cohorts (Fig. G2) and heterozygosity (Fig. G3).

**Table G1**. Results of linear models testing whether population structure (site; LM.G.site), environmental variation during birth year (cohort; LM.G.cohort), site+cohort (LM.G.site.cohort) or site+cohort+site*cohort interaction (LM.G.site*cohort) explain difference in juvenile growth residuals of Murrumbidgee Macquarie perch. LM.G.site: Growth_residuals ~site; LM.G.cohort: Growth_residuals ~cohort; LM.G.site.cohort: Growth_residuals ~site+cohort, LM.G.site*cohort: Growth_residuals~ site*cohort; LM.G.best.H: Growth_residuals~ site*cohort + PHt. P<0.05 are in red. Results of posthoc Tukey tests in Tables G2 (LM.G.site) and G3 (LM.G.cohort), plots in Fig. G1, G2 and Figure 4 of the main text.

| **Model** | **Term** | **Estimate** | **Std.Error** | **t value** | **Pr(>\|t\|)** | **Adjusted R-squared** | **P-value** |
| --- | --- | --- | --- | --- | --- | --- | --- |
| LM.G.site | (Intercept) | -19.844 | 6.587 | -3.013 | 0.003 | 0.233 | 0.000 |
|  | Site2 | 25.528 | 6.702 | 3.809 | 0.000 |  |  |
|  | Site3 | 25.247 | 6.848 | 3.687 | 0.000 |  |  |
|  | Site4 | 15.422 | 7.873 | 1.959 | 0.051 |  |  |
|  | Site5 | 6.199 | 7.088 | 0.875 | 0.383 |  |  |
|  | Site6 | 24.897 | 7.724 | 3.224 | 0.001 |  |  |
|  | Site7 | 21.569 | 13.173 | 1.637 | 0.103 |  |  |
|  | Site8 | 16.927 | 6.705 | 2.525 | 0.012 |  |  |
|  | Site9 | 6.374 | 7.873 | 0.810 | 0.419 |  |  |
| LM.G.cohort | (Intercept) | 10.775 | 2.199 | 4.900 | 0.000 | 0.116 | 0.000 |
|  | Cohort2019 | -14.306 | 2.930 | -4.883 | 0.000 |  |  |
|  | Cohort2020 | -10.476 | 2.424 | -4.321 | 0.000 |  |  |
|  | Cohort2021 | -22.674 | 5.461 | -4.152 | 0.000 |  |  |
|  | Cohort2022 | -15.098 | 3.163 | -4.773 | 0.000 |  |  |
| LM.G.site.cohort | (Intercept) | -13.005 | 6.730 | -1.932 | 0.055 | 0.297 | 0.000 |
|  | Site2 | 25.118 | 6.499 | 3.865 | 0.000 |  |  |
|  | Site3 | 25.585 | 6.559 | 3.901 | 0.000 |  |  |
|  | Site4 | 15.422 | 7.536 | 2.046 | 0.042 |  |  |
|  | Site5 | 7.959 | 6.829 | 1.165 | 0.245 |  |  |
|  | Site6 | 27.524 | 7.562 | 3.640 | 0.000 |  |  |
|  | Site7 | 25.773 | 12.864 | 2.004 | 0.046 |  |  |
|  | Site8 | 18.129 | 6.464 | 2.804 | 0.005 |  |  |
|  | Site9 | 9.776 | 7.672 | 1.274 | 0.204 |  |  |
|  | Cohort2019 | -11.616 | 2.659 | -4.369 | 0.000 |  |  |
|  | Cohort2020 | -6.839 | 2.353 | -2.906 | 0.004 |  |  |
|  | Cohort2021 | -16.646 | 5.172 | -3.219 | 0.001 |  |  |
|  | Cohort2022 | -11.043 | 3.279 | -3.368 | 0.001 |  |  |

| **Model** | | **Term** | | **Estimate** | | **Std.Error** | | **t value** | | **Pr(>\|t\|)** | | **Adjusted R-squared** | | **P-value** | |
| --- | --- | --- | --- | --- | --- | --- | --- | --- | --- | --- | --- | --- | --- | --- | --- |
| LM.G.site*cohort | | (Intercept) | | 1.636 | | 14.042 | | 0.117 | | 0.907 | | 0.3524 | | 0.000 | |
|  | | Site2 | | 9.333 | | 14.204 | | 0.657 | | 0.512 | |  | |  | |
|  | | Site3 | | 6.034 | | 17.522 | | 0.344 | | 0.731 | |  | |  | |
|  | | Site4 | | 15.422 | | 7.232 | | 2.132 | | 0.034 | |  | |  | |
|  | | Site5 | | 11.916 | | 6.765 | | 1.761 | | 0.080 | |  | |  | |
|  | | Site6 | | 11.830 | | 13.995 | | 0.845 | | 0.399 | |  | |  | |
|  | | Site7 | | 15.659 | | 16.844 | | 0.930 | | 0.354 | |  | |  | |
|  | | Site8 | | 8.878 | | 13.375 | | 0.664 | | 0.507 | |  | |  | |
|  | | Site9 | | 8.574 | | 8.004 | | 1.071 | | 0.285 | |  | |  | |
|  | | Cohort2019 | | -23.689 | | 6.050 | | -3.915 | | 0.000 | |  | |  | |
|  | | Cohort2020 | | -21.480 | | 12.672 | | -1.695 | | 0.091 | |  | |  | |
|  | | Cohort2021 | | -32.138 | | 13.713 | | -2.344 | | 0.020 | |  | |  | |
|  | | Cohort2022 | | -15.570 | | 4.827 | | -3.226 | | 0.001 | |  | |  | |
|  | | Site2:Cohort2019 | | 16.332 | | 6.751 | | 2.419 | | 0.016 | |  | |  | |
|  | | Site3:Cohort2019 | | 21.834 | | 14.190 | | 1.539 | | 0.125 | |  | |  | |
|  | | Site4:Cohort2019 | | NA | | NA | | NA | | NA | |  | |  | |
|  | | Site5:Cohort2019 | | -13.307 | | 13.617 | | -0.977 | | 0.329 | |  | |  | |
|  | | Site6:Cohort2019 | | NA | | NA | | NA | | NA | |  | |  | |
|  | | Site7:Cohort2019 | | NA | | NA | | NA | | NA | |  | |  | |
|  | | Site8:Cohort2019 | | NA | | NA | | NA | | NA | |  | |  | |
|  | | Site9:Cohort2019 | | NA | | NA | | NA | | NA | |  | |  | |
|  | | Site2:Cohort2020 | | 15.032 | | 12.973 | | 1.159 | | 0.248 | |  | |  | |
|  | | Site3:Cohort2020 | | 19.722 | | 16.545 | | 1.192 | | 0.234 | |  | |  | |
|  | | Site4:Cohort2020 | | NA | | NA | | NA | | NA | |  | |  | |
|  | | Site5:Cohort2020 | | NA | | NA | | NA | | NA | |  | |  | |
|  | | Site6:Cohort2020 | | 24.996 | | 13.995 | | 1.786 | | 0.075 | |  | |  | |
|  | | Site7:Cohort2020 | | NA | | NA | | NA | | NA | |  | |  | |
|  | | Site8:Cohort2020 | | 8.188 | | 12.025 | | 0.681 | | 0.497 | |  | |  | |
|  | | Site9:Cohort2020 | | NA | | NA | | NA | | NA | |  | |  | |
|  | | Site2:Cohort2021 | | -7.493 | | 17.391 | | -0.431 | | 0.667 | |  | |  | |
|  | | Site3:Cohort2021 | | 9.963 | | 20.191 | | 0.493 | | 0.622 | |  | |  | |
|  | | Site4:Cohort2021 | | NA | | NA | | NA | | NA | |  | |  | |
|  | | Site5:Cohort2021 | | NA | | NA | | NA | | NA | |  | |  | |
|  | | Site6:Cohort2021 | | NA | | NA | | NA | | NA | |  | |  | |
|  | | Site7:Cohort2021 | | NA | | NA | | NA | | NA | |  | |  | |
|  | | Site8:Cohort2021 | | 29.438 | | 14.988 | | 1.964 | | 0.051 | |  | |  | |
|  | | Site9:Cohort2021 | | NA | | NA | | NA | | NA | |  | |  | |
|  | | Site2:Cohort2022 | | NA | | NA | | NA | | NA | |  | |  | |
|  | | Site3:Cohort2022 | | NA | | NA | | NA | | NA | |  | |  | |
|  | | Site4:Cohort2022 | | NA | | NA | | NA | | NA | |  | |  | |
|  | | Site5:Cohort2022 | | NA | | NA | | NA | | NA | |  | |  | |
|  | | Site6:Cohort2022 | | NA | | NA | | NA | | NA | |  | |  | |
|  | | Site7:Cohort2022 | | NA | | NA | | NA | | NA | |  | |  | |
|  | | Site8:Cohort2022 | | NA | | NA | | NA | | NA | |  | |  | |
|  | | Site9:Cohort2022 | | NA | | NA | | NA | | NA | |  | |  | |
| **Model** | **Term** | | **Estimate** | | **Std.Error** | | **t value** | | **Pr(>\|t\|)** | | **Adjusted R-squared** | | **P-value** | |  |
| LM.G.best.H | (Intercept) | | 3.745 | | 14.194 | | 0.264 | | 0.792 | | 0.353 | | 0.00 | |  |
|  | PHt_Murr | | -33.403 | | 32.877 | | -1.016 | | 0.311 | |  | |  | |  |
|  | Site2 | | 10.390 | | 14.241 | | 0.730 | | 0.466 | |  | |  | |  |
|  | Site3 | | 6.781 | | 17.536 | | 0.387 | | 0.699 | |  | |  | |  |
|  | Site4 | | 15.410 | | 7.231 | | 2.131 | | 0.034 | |  | |  | |  |
|  | Site5 | | 13.288 | | 6.898 | | 1.926 | | 0.055 | |  | |  | |  |
|  | Site6 | | 13.184 | | 14.057 | | 0.938 | | 0.349 | |  | |  | |  |
|  | Site7 | | 17.331 | | 16.923 | | 1.024 | | 0.307 | |  | |  | |  |
|  | Site8 | | 10.622 | | 13.483 | | 0.788 | | 0.432 | |  | |  | |  |
|  | Site9 | | 9.819 | | 8.097 | | 1.213 | | 0.226 | |  | |  | |  |
|  | Cohort2019 | | -23.547 | | 6.052 | | -3.891 | | 0.000 | |  | |  | |  |
|  | Cohort2020 | | -21.017 | | 12.679 | | -1.658 | | 0.099 | |  | |  | |  |
|  | Cohort2021 | | -31.444 | | 13.729 | | -2.290 | | 0.023 | |  | |  | |  |
|  | Cohort2022 | | -15.398 | | 4.829 | | -3.188 | | 0.002 | |  | |  | |  |
|  | Site2:Cohort2019 | | 15.776 | | 6.773 | | 2.329 | | 0.021 | |  | |  | |  |
|  | Site3:Cohort2019 | | 22.865 | | 14.225 | | 1.607 | | 0.109 | |  | |  | |  |
|  | Site4:Cohort2019 | | NA | | NA | | NA | | NA | |  | |  | |  |
|  | Site5:Cohort2019 | | -13.568 | | 13.618 | | -0.996 | | 0.320 | |  | |  | |  |
|  | Site6:Cohort2019 | | NA | | NA | | NA | | NA | |  | |  | |  |
|  | Site7:Cohort2019 | | NA | | NA | | NA | | NA | |  | |  | |  |
|  | Site8:Cohort2019 | | NA | | NA | | NA | | NA | |  | |  | |  |
|  | Site9:Cohort2019 | | NA | | NA | | NA | | NA | |  | |  | |  |
|  | Site2:Cohort2020 | | 14.878 | | 12.973 | | 1.147 | | 0.253 | |  | |  | |  |
|  | Site3:Cohort2020 | | 20.023 | | 16.546 | | 1.210 | | 0.227 | |  | |  | |  |
|  | Site4:Cohort2020 | | NA | | NA | | NA | | NA | |  | |  | |  |
|  | Site5:Cohort2020 | | NA | | NA | | NA | | NA | |  | |  | |  |
|  | Site6:Cohort2020 | | 25.431 | | 14.000 | | 1.816 | | 0.071 | |  | |  | |  |
|  | Site7:Cohort2020 | | NA | | NA | | NA | | NA | |  | |  | |  |
|  | Site8:Cohort2020 | | 7.792 | | 12.031 | | 0.648 | | 0.518 | |  | |  | |  |
|  | Site9:Cohort2020 | | NA | | NA | | NA | | NA | |  | |  | |  |
|  | Site2:Cohort2021 | | -8.737 | | 17.432 | | -0.501 | | 0.617 | |  | |  | |  |
|  | Site3:Cohort2021 | | 10.327 | | 20.193 | | 0.511 | | 0.610 | |  | |  | |  |
|  | Site4:Cohort2021 | | NA | | NA | | NA | | NA | |  | |  | |  |
|  | Site5:Cohort2021 | | NA | | NA | | NA | | NA | |  | |  | |  |
|  | Site6:Cohort2021 | | NA | | NA | | NA | | NA | |  | |  | |  |
|  | Site7:Cohort2021 | | NA | | NA | | NA | | NA | |  | |  | |  |
|  | Site8:Cohort2021 | | 28.457 | | 15.018 | | 1.895 | | 0.059 | |  | |  | |  |
|  | Site9:Cohort2021 | | NA | | NA | | NA | | NA | |  | |  | |  |
|  | Site2:Cohort2022 | | NA | | NA | | NA | | NA | |  | |  | |  |
|  | Site3:Cohort2022 | | NA | | NA | | NA | | NA | |  | |  | |  |
|  | Site4:Cohort2022 | | NA | | NA | | NA | | NA | |  | |  | |  |
|  | Site5:Cohort2022 | | NA | | NA | | NA | | NA | |  | |  | |  |
|  | Site6:Cohort2022 | | NA | | NA | | NA | | NA | |  | |  | |  |
|  | Site7:Cohort2022 | | NA | | NA | | NA | | NA | |  | |  | |  |
|  | Site8:Cohort2022 | | NA | | NA | | NA | | NA | |  | |  | |  |
|  | Site9:Cohort2022 | | NA | | NA | | NA | | NA | |  | |  | |  |

**Table G2.** Results of posthoc Tukey test for model LM.G.site: comparing growth residuals between Murrumbidgee sites. Diff- difference between the means of the two groups being compared (value >0 indicates that the first group has a higher mean than the second group); lwr- the lower bound of the confidence interval for the difference between the group; upr- the upper bound of the confidence interval for the difference between the group means; p adj- P-value adjusted for multiple comparisons using the Tukey method.

| **Pair of sites** | **diff** | **lwr** | **upr** | **p adj** |
| --- | --- | --- | --- | --- |
| Sites 2-1 | 25.5 | 4.6 | 46.5 | 0.005 |
| Sites 3-1 | 25.2 | 3.8 | 46.7 | 0.008 |
| Sites 4-1 | 15.4 | -9.2 | 40.1 | 0.574 |
| Sites 5-1 | 6.2 | -16.0 | 28.4 | 0.994 |
| Sites 6-1 | 24.9 | 0.7 | 49.1 | 0.038 |
| Sites 7-1 | 21.6 | -19.7 | 62.8 | 0.783 |
| Sites 8-1 | 16.9 | -4.1 | 37.9 | 0.225 |
| Sites 9-1 | 6.4 | -18.3 | 31.0 | 0.997 |
| Sites 3-2 | -0.3 | -7.3 | 6.8 | 1.000 |
| Sites 4-2 | -10.1 | -24.1 | 3.9 | 0.375 |
| Sites 5-2 | -19.3 | -28.4 | -10.3 | 0.000 |
| Sites 6-2 | -0.6 | -13.8 | 12.6 | 1.000 |
| Sites 7-2 | -4.0 | -39.9 | 32.0 | 1.000 |
| Sites 8-2 | -8.6 | -14.1 | -3.1 | 0.000 |
| Sites 9-2 | -19.2 | -33.2 | -5.1 | 0.001 |
| Sites 4-3 | -9.8 | -24.5 | 4.9 | 0.483 |
| Sites 5-3 | -19.0 | -29.1 | -9.0 | 0.000 |
| Sites 6-3 | -0.4 | -14.3 | 13.6 | 1.000 |
| Sites 7-3 | -3.7 | -39.9 | 32.5 | 1.000 |
| Sites 8-3 | -8.3 | -15.4 | -1.3 | 0.008 |
| Sites 9-3 | -18.9 | -33.6 | -4.2 | 0.003 |
| Sites 5-4 | -9.2 | -25.0 | 6.6 | 0.663 |
| Sites 6-4 | 9.5 | -9.0 | 28.0 | 0.801 |
| Sites 7-4 | 6.1 | -32.0 | 44.3 | 1.000 |
| Sites 8-4 | 1.5 | -12.5 | 15.6 | 1.000 |
| Sites 9-4 | -9.0 | -28.1 | 10.0 | 0.862 |
| Sites 6-5 | 18.7 | 3.6 | 33.7 | 0.004 |
| Sites 7-5 | 15.4 | -21.3 | 52.0 | 0.927 |
| Sites 8-5 | 10.7 | 1.6 | 19.8 | 0.008 |
| Sites 9-5 | 0.2 | -15.6 | 16.0 | 1.000 |
| Sites 7-6 | -3.3 | -41.2 | 34.5 | 1.000 |
| Sites 8-6 | -8.0 | -21.2 | 5.3 | 0.623 |
| Sites 9-6 | -18.5 | -37.0 | 0.0 | 0.049 |
| Sites 8-7 | -4.6 | -40.6 | 31.3 | 1.000 |
| Sites 9-7 | -15.2 | -53.4 | 23.0 | 0.945 |
| Sites 9-8 | -10.6 | -24.6 | 3.5 | 0.317 |

**Table G3.** Results of posthoc Tukey test for model LM.G.cohort: comparing growth residuals between Murrumbidgee cohorts. Diff- difference between the means of the two groups being compared (value >0 indicates that the first group has a higher mean than the second group); lwr- the lower bound of the confidence interval for the difference between the group; upr- the upper bound of the confidence interval for the difference between the group means; p adj- P-value adjusted for multiple comparisons using the Tukey method.

| **Pair of cohorts** | **diff** | **lwr** | **upr** | **P adj** |
| --- | --- | --- | --- | --- |
| 2019-2018 | -14.3 | -22.4 | -6.3 | 0.000 |
| 2020-2018 | -10.5 | -17.1 | -3.8 | 0.000 |
| 2021-2018 | -22.7 | -37.7 | -7.7 | 0.000 |
| 2022-2018 | -15.1 | -23.8 | -6.4 | 0.000 |
| 2020-2019 | 3.8 | -2.2 | 9.8 | 0.405 |
| 2021-2019 | -8.4 | -23.1 | 6.4 | 0.524 |
| 2022-2019 | -0.8 | -9.0 | 7.4 | 0.999 |
| 2021-2020 | -12.2 | -26.2 | 1.8 | 0.121 |
| 2022-2020 | -4.6 | -11.5 | 2.2 | 0.345 |
| 2022-2021 | 7.6 | -7.5 | 22.7 | 0.641 |
|  |  |  |  |  |

**Figure G1.** Size-at-age residuals by Murrumbidgee site (cohorts pooled; LM.G.site in Table G1). Juveniles from sites 1, 5, 8 and 9 are significantly smaller than those from sites 2 and 3; juveniles from site 5 are smaller than those from sites 6 and 8; and juveniles from site 9 are smaller than those from site 6 (P<0.05; Tukey tests in Table G2).


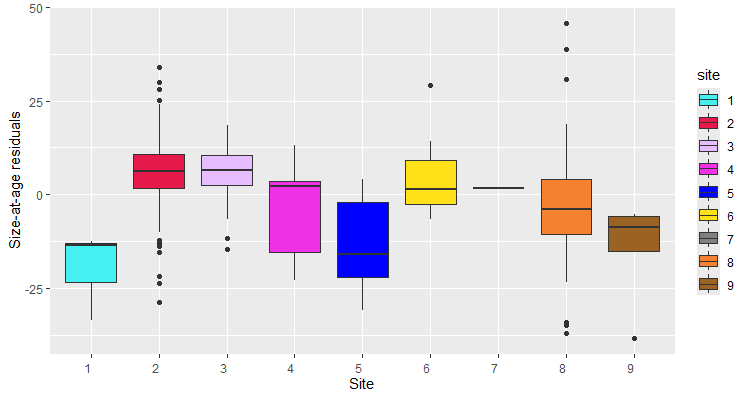


**Figure G2.** Size-at-age residuals residuals of Murrumbidgee Macquarie perch for five juvenile cohorts (sites pooled). Cohort 2018 had significantly larger residuals compared to other cohorts (Turkey test P<0.001; Table G3).

**
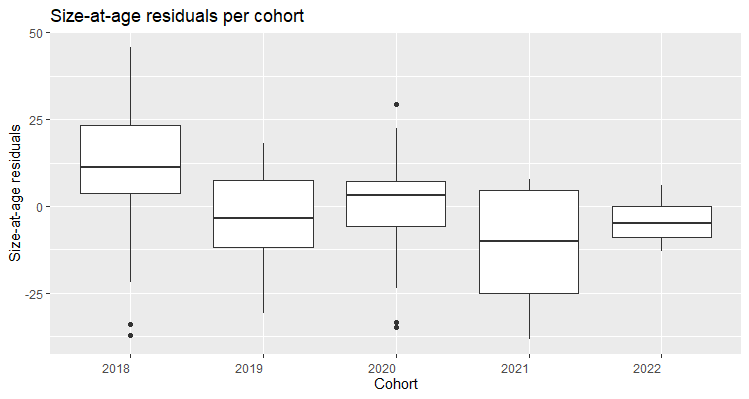
**

**Figure G3.** Scatterplot of growth residuals vs individual heterozygosity of Murrumbidgee Macquarie perch, with points coloured by cohort (top) or sites (bottom).


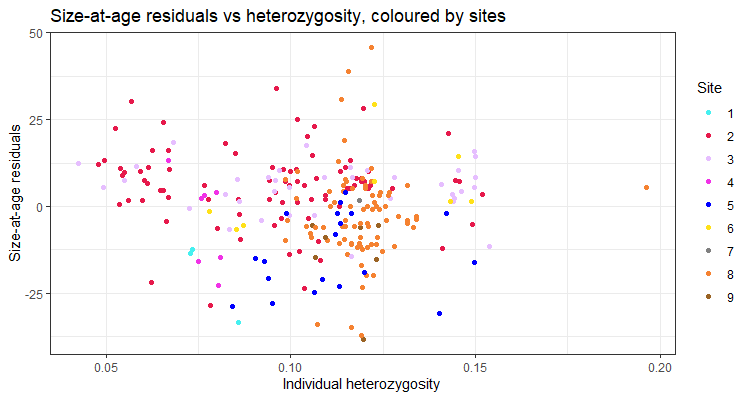


# **Appendix H.** Detailed recommendations for genetic management of Murrumbidgee Macquarie perch population.

Providing best-practice management to the spatially large but genetically compromised Murrumbidgee Macquarie perch population is essential to ensure that it persists, that its unique genetic diversity is not lost through genetic drift, and that the overall recovery of this threatened species is promoted through gene exchange among the major catchments of the Murray-Darling Basin. This includes:

- Improving connectivity among sites within the upper Murrumbidgee River to reduce inbreeding and genetic drift should be of the highest management priority. This could be achieved by

1. providing adequate flow releases from Tantangara Reservoir, i.e. at levels which would demonstrably permit natural dispersal in upstream and downstream directions, and
2. conducting translocations among river reaches to reduce inbreeding at local scales. Translocating fish to upstream sites 1-3, where numbers of breeders and genetic diversity were lowest and inbreeding strongest, is especially important in order to maintain genetic health of the recruitment reach. Translocating fish from sites 10-12 (outside of the recruitment reach) to the upstream sites should be considered. Based on the observed population genetic structure, four major genetic groups should be reconnected: sites 1-3, site 4, site 5 and sites 7-9. Given lack of gene flow from sites 1-3 to sites 7-9, some translocations in both directions should be also considered, to reduce chance loss of genetic variation. If these translocations are successful, monitoring in 7-10 years after translocations should observe genetic structure reduced or absent.

- Continuing wild-to-wild translocations among populations to increase genetic diversity is also of highest priority. Genetic risk assessment for the species suggests that negative consequences of such admixture are highly unlikely (Pavlova et al. 2017). Sources for Murrumbidgee include:

1. Cataract Reservoir, with ancestry from the lower Murrumbidgee, and
2. Genetically diverse and divergent populations from other catchments, including Yarra and Ovens Rivers (both translocated populations originated from the Murray River catchment) or Abercrombie River (Lachlan River catchment). Augmenting a relatively isolated site (e.g. site 4) with cross-catchment translocations would make it possible to monitor survival and reproductive success of fish with different ancestries, as has been done elsewhere (Lutz et al. 2022) to ensure such admixture is overall beneficial for the local population before attempting translocations to other Murrumbidgee sites.

- Stocking with genetically diverse (admixed) offspring should be considered.

1. Given the low genetic diversity of other populations in the Murrumbidgee catchment (Pavlova et al. 2017), captive breeding of admixed offspring of parents from different low-diversity Murrumbidgee Catchment populations, such as Cotter River/Reservoir or Adjungbilly Creek guarantees admixture without relying on mate choice and other dynamics in the wild leading to the desired crosses. Such captive-bred inter-population crosses will be more genetically diverse than the parents.
2. Cross-catchment crosses, where parents of Murrumbidgee Catchment ancestry (i.e. Murrumbidgee River, Cotter River/Reservoir, Adjungbilly Creek) are crossed with parents from other catchments (e.g. Yarra River, Ovens River, Abercrombie Rivers).

- Genetic monitoring of Macquarie perch cohorts in the Murrumbidgee should continue, to evaluate the impact of interventions and enable adjustments. Success of genetic management could be ascertained by signatures of reduced population structure within Murrumbidgee and increase in genetic diversity per site.
- If the upper Murrumbidgee River population is used as a source of individuals for genetic augmentation of other populations (e.g. the Cotter River, Pavlova et al. 2024), it should be included as one of two or more sources, to increase/maximise genetic diversity. Any harvested individuals should be replaced by migrants from other locations, and effects on source populations of harvesting for translocation should be monitored to ensure the number of breeders does not decline as a result (Mitchell et al. 2022).

**References:**

de Jong, M. J., de Jong, J. F., Hoelzel, A. R., & Janke, A. (2021). SambaR: An R package for fast, easy and reproducible population‐genetic analyses of biallelic SNP data sets. *Molecular Ecology Resources* 21, 1369-1379.

Frichot, E., Mathieu, F., Trouillon, T., Bouchard, G., & François, O. (2014). Fast and efficient estimation of individual ancestry coefficients. *Genetics* 196, 973-983.

Lutz, M. L., Sunnucks, P., Chapple, D. G., Gilligan, D., Lintermans, M., & Pavlova, A. (2022). Strong bidirectional gene flow between fish lineages separated for over a 100,000 years. *Conservation Genetics* 23, 1105–1113.

Mitchell, W. F., Boulton, R. L., Sunnucks, P., & Clarke, R. H. (2022). Are we adequately assessing the demographic impacts of harvesting for wild‐sourced conservation translocations? *Conservation Science and Practice* 4, e569.

Mussmann, S. M., Douglas, M. R., Chafin, T. K., & Douglas, M. E. (2019). BA3‐SNPs: Contemporary migration reconfigured in BayesAss for next‐generation sequence data. *Methods in Ecology and Evolution* 10, 1808-1813.

Paradis, E. & Schliep, K. (2018). ape 5.0: an environment for modern phylogenetics and evolutionary analyses in R. *Bioinformatics* 35, 526-528.

Pavlova, A., Beheregaray, L. B., Coleman, R., Gilligan, D., Harrisson, K. A., Ingram, B. A., . . . Sunnucks, P. (2017). Severe consequences of habitat fragmentation on genetic diversity of an endangered Australian freshwater fish: a call for assisted gene flow. *Evolutionary Applications* 10, 531–550.

Pavlova, A., Harrisson, K. A., Turakulov, R. I., Lee, Y. P., Ingram, B., Gilligan, D., . . . Gan, H. M. (2022). Labile sex chromosomes in the Australian freshwater fish family Percichthyidae. *Molecular Ecology Resources* 22, 1639-1655.

Pavlova, A., Schneller, N., Lintermans, M., Beitzel, M., Robledo-Ruiz, D., & Sunnucks, P. (2024). Planning and implementing genetic rescue of an endangered freshwater fish population in a regulated river, where low flow reduces breeding opportunities and may trigger inbreeding depression. *Evolutionary Applications* 17, e13679.

Pembleton, L. W., Cogan, N. O. I., & Forster, J. W. (2013). StAMPP: an R package for calculation of genetic differentiation and structure of mixed-ploidy level populations. *Molecular Ecology Resources* 13, 946-952.

Robledo-Ruiz, D. A., Pavlova, A., Clarke, R. H., Magrath, M. J., Quin, B., Harrisson, K. A., . . . Sunnucks, P. (2022). A novel framework for evaluating *in-situ* breeding management strategies in endangered populations. *Molecular Ecology Resources* 22, 239-253.

Zhang, C., Dong, S.-S., Xu, J.-Y., He, W.-M., & Yang, T.-L. (2018). PopLDdecay: a fast and effective tool for linkage disequilibrium decay analysis based on variant call format files. *Bioinformatics* 35, 1786-1788.
